# Extraction Matrix Shapes the Efficacy of Gegen Qinlian Decoction in DSS-Induced Colitis: A Preclinical Systematic Review and Meta-Analysis

**DOI:** 10.3390/ph19020277

**Published:** 2026-02-06

**Authors:** Carlos R. Montes-de-Oca-Saucedo, Bruno Briceño-Villardaga, Sebastián R. Fuentes-Salinas, Adolfo Soto-Domínguez

**Affiliations:** Department of Histology, Faculty of Medicine, Universidad Autónoma de Nuevo León (UANL), Monterrey 64460, Nuevo León, Mexico; bruno.bricenov@uanl.edu.mx (B.B.-V.); sebastian.fuentess@uanl.edu.mx (S.R.F.-S.); adolfo.sotodmn@uanl.edu.mx (A.S.-D.)

**Keywords:** Gegen Qinlian Decoction, DSS-induced colitis, ulcerative colitis, systematic review, meta-analysis, extraction matrix

## Abstract

**Background:** Ulcerative colitis (UC) is a chronic inflammatory disease marked by mucosal injury and immune dysregulation. Gegen Qinlian Decoction (GQD) shows therapeutic potential, but extraction-dependent reproducibility remains unclear. **Methods:** We systematically searched PubMed, Scopus, and Web of Science for studies evaluating GQD aqueous decoctions or ethanolic extracts in DSS-induced colitis. Main outcome: Disease Activity Index (DAI). Key additional outcomes: colon length and histological injury; cytokines and microbiota were also assessed. Random-effects models with Hartung–Knapp–Sidik–Jonkman adjustment, subgroup analyses, and exploratory dose–response meta-regression were applied. **Results:** Eight studies were included (aqueous: 5; ethanolic: 3; 209 mice). GQD significantly improved DAI (SMD −2.17; *p* < 0.00001; I^2^ = 43%), colon length (MD 1.18 cm; *p* < 0.00001; I^2^ = 88%), and histological injury (SMD −3.02; *p* < 0.0001; I^2^ = 51%). For DAI, both preparations favored GQD, with absent heterogeneity in aqueous studies (I^2^ = 0%) vs. substantial variability in ethanolic extracts (I^2^ = 75%). For histology, subgroup differences suggested a larger effect size with ethanolic extracts, with higher heterogeneity (I^2^ = 60% vs. 0%). In the aqueous subset, GQD reduced inflammatory markers and increased microbial diversity. Dose–response meta-regression was performed within the aqueous subset as an exploratory analysis. **Conclusions:** Aqueous decoctions showed the most reproducible profile across key endpoints, whereas ethanolic extracts were more variable despite a larger histology point estimate, indicating that the extraction matrix meaningfully influences translational consistency in preclinical research.

## 1. Introduction

Ulcerative colitis (UC) is a chronic, relapsing inflammatory bowel disease (IBD) that primarily affects the colonic mucosa and is characterized by abdominal pain, bloody diarrhea, and a marked impairment in quality of life [1]. In North America, UC affects approximately 1.5 million individuals [2]. Although incidence rates have stabilized in several Western regions, overall prevalence continues to rise, thereby increasing the long-term disease burden [3]. This clinical impact is further compounded by substantial direct and indirect healthcare costs [4].

Current pharmacological management remains imperfect. Corticosteroids are widely used for induction of remission; however, up to 38% of patients relapse during tapering or become steroid-dependent [5]. In patients with moderate-to-severe disease, biologic and small-molecule therapies have significantly improved outcomes, yet approximately 30–40% of individuals fail to achieve sustained clinical remission or experience secondary loss of response [6]. As a result, the therapeutic landscape has become increasingly complex, with multiple agents targeting distinct pathways. Recent large-scale network meta-analyses underscore both the progress achieved and the persistence of important unmet clinical needs [7].

Against this background, Gegen Qinlian Decoction (GQD) has attracted growing interest in controlled experimental settings as a potential therapeutic strategy. GQD is a classical traditional Chinese medicine formula originating from the *Shang Han Lun* (Treatise on Febrile Diseases), historically used for gastrointestinal syndromes such as diarrhea/diarrhea with fever, which provides a traditional rationale for its frequent evaluation in experimental colitis models [8,9]. GQD is a multi-herb formulation composed of *Pueraria lobata*, *Scutellaria baicalensis*, *Coptis chinensis*, and *Glycyrrhiza uralensis*. Comprehensive pharmacological studies have characterized its major bioactive constituents and highlighted the complex interactions inherent to multicomponent formulations [8,9]. Accumulating mechanistic evidence suggests that GQD exerts protective effects on the intestinal mucosa by modulating gut microbiota composition and activating immune pathways involved in barrier maintenance and repair [10].

Pharmacokinetic studies further indicate that different GQD preparations can substantially influence the systemic and colonic bioavailability of its constituents, and both traditional aqueous decoctions and modern ethanolic extracts are commonly employed in preclinical research [11]. Although clinical meta-analyses support the efficacy of GQD as an adjunct to mesalazine in patients with UC [12], a comprehensive preclinical synthesis assessing its standalone efficacy has not yet been performed, and sources of methodological variability across experimental studies remain insufficiently explored [13].

Importantly, herbal formulations such as GQD pose distinct challenges for evidence synthesis. Their pharmacological behavior depends not only on the presence of individual bioactive compounds but also on the extraction matrix, which governs multicomponent interactions, physicochemical stability, and bioavailability [8,11]. Experimental studies have utilized both aqueous and ethanolic preparations in preclinical models of colitis [11,13], yet the extent to which extraction methods influence pharmacological reproducibility and consistency has not been systematically examined. This issue is particularly relevant for multicomponent therapies, where variability in preparation may obscure true biological effects and complicate translational interpretation. Accordingly, we conducted a systematic review and meta-analysis to evaluate the pharmacological efficacy of GQD in preclinical colitis models, specifically stratifying analyses by extraction matrix (aqueous vs. ethanolic) to assess the impact of the formulation matrix on therapeutic reproducibility.

## 2. Materials and Methods

### 2.1. Protocol and Registration

This systematic review and meta-analysis was conducted in accordance with the PRISMA 2020 guidelines [14]. The protocol was prospectively registered on the Open Science Framework [15] and subsequently on INPLASY (Registration INPLASY202590077) [16]. All stages of study selection, data extraction, and synthesis were performed according to the pre-specified protocol to ensure methodological transparency and reproducibility.

### 2.2. Eligibility Criteria

Eligible studies were in vivo mouse experiments using dextran sulfate sodium (DSS) to induce colitis and evaluating traditional aqueous decoction or ethanolic extract preparations of the four-herb Gegen Qinlian Decoction (GQD). To minimize variability due to pharmaceutical formulation, only unmodified GQD formulations were included. For terminology consistency, we defined modified formula as GQD variants that add herbal ingredients beyond the classic four-herb composition (>4 herbs; e.g., six-herb versions), whereas different formulation refers to non-traditional/engineered preparations of the classic four-herb GQD (e.g., granules, reformulated/fractionated products, or nanoparticle-enhanced delivery systems). Studies employing concentrated, reformulated, fractionated, or engineered nanoparticle-enhanced preparations were excluded. Comparator groups consisted of DSS-exposed animals receiving no active pharmacological treatment. Studies were required to report at least one eligible outcome for quantitative synthesis, with DAI defined as the main outcome.

For quantitative synthesis, the primary comparison was GQD-treated DSS mice versus DSS-only (vehicle/no active pharmacological treatment) controls. When studies reported multiple GQD dose arms sharing a single DSS control, each eligible dose arm was extracted. We accounted for the non-independence of these comparisons as described in the Section 2.8. Additional arms (e.g., healthy/blank controls or positive-drug controls) were not used in the pooled comparisons. Only peer-reviewed original full-text articles published in English were included.

### 2.3. Search Strategy and Data Extraction

We systematically searched PubMed, Scopus, and Web of Science from inception to July 2025 using both controlled vocabulary and free-text terms related to “Gegen Qinlian Decoction” and “dextran sulfate sodium colitis”. Full search strategies for all databases are provided in Supplementary Table S1. Two reviewers (C.R.M.-d.-O.-S. and B.B.-V.) independently screened all records using Rayyan [17]. Disagreements were resolved through consensus or consultation with a third reviewer (A.S.-D.). Full-text articles excluded after eligibility assessment, along with reasons for exclusion, are reported in Supplementary Table S5. Reference lists of included articles and related reviews were screened to identify potentially eligible additional records. Two reviewers (C.R.M.-d.-O.-S. and S.R.F.-S.) independently extracted data on study design, animal strain and sex, DSS concentration, GQD preparation method, dose, treatment timing, and all reported outcomes. When numerical values were available only in graphical form, data were digitized using WebPlotDigitizer v4.6 [18]. If essential data were missing or unclear, corresponding authors were contacted; in the absence of clarification, studies were analyzed using the available information.

To assess chemical and pharmacological reproducibility, we explicitly extracted data regarding the extraction matrix (solvent type), phytochemical characterization (e.g., HPLC/UPLC fingerprinting and marker quantification), and endotoxin control reporting (e.g., endotoxin-free certification or LAL testing), when available. Herbal intervention reporting completeness was evaluated using a 14-item Reporting Characteristics Score (RCS-14), based on strict evidence-direct reporting from the main text, figures, and Supplementary Materials. The RCS-14 domains were adapted from PRISMA-CHM 2020 reporting items relevant to Chinese herbal medicine intervention characterization (e.g., botanical identity, extraction processing, chemical quality control, and administration details) [19].

### 2.4. Outcomes

The main outcome was the Disease Activity Index (DAI), as reported by each study. Additional outcomes were colon length (CL), histological injury score (HS), myeloperoxidase (MPO) activity, body weight change, inflammatory cytokines (TNF-α, IL-6, IL-1β), and microbial diversity measures (e.g., Shannon index). Body weight change was extracted as percentage change (percentage points), typically relative to baseline. For directionality, lower DAI/HS/cytokine/MPO levels and less body weight loss indicated improvement, whereas longer colon length indicated improvement.

### 2.5. Subgroup and Dose–Response Analyses

Several studies included multiple GQD dosing arms sharing a single DSS-only control. Therefore, each eligible dose arm was extracted as a separate comparison and assigned to its corresponding extraction subgroup (aqueous decoction vs. ethanolic extract). To minimize unit-of-analysis errors arising from correlated effect sizes, non-independence was addressed as described in the Section 2.8. Subgroup analyses were primarily stratified by extraction solvent to evaluate the impact of the preparation matrix on pharmacological reproducibility. Dose values were extracted as reported (g/kg). When explicitly stated, we recorded whether dosing referred to crude-drug equivalents or to dried extract mass. Because extraction yields (drug-to-extract ratios) and dosing bases were inconsistently reported, we did not convert doses to a unified raw-material-equivalent scale. For this reason, dose-related analyses are presented as exploratory and should be interpreted cautiously as reflecting reported dosing practice rather than standardized potency. Dose-tier subgroup analyses and continuous dose–response meta-regression were restricted to the aqueous subset. Given heterogeneity in assays, timepoints, and reporting, particularly among the limited ethanolic-extract studies (n = 3), quantitative pooling for secondary mechanistic endpoints was restricted to outcomes with sufficient comparability, and the remaining endpoints were summarized narratively.

Dose–response patterns were explored only when supported by adequate within-study dose variation and sufficient comparability. Two complementary approaches were used. First, aqueous comparisons were grouped into lower-range (<7.5 g/kg) versus higher-range (≥7.5 g/kg) reported-dose categories. This cutpoint was pre-specified and informed by approximate clinical dose-scaling estimates (21–40 g/day raw herb ≈ 3.5–6.5 g/kg in mice, scaled by body surface area) [20,21], using 7.5 g/kg as a pragmatic threshold near the upper bound of this range. Given that doses were analyzed as reported and were not harmonized to a single raw-material-equivalent scale, this stratification is intended as exploratory rather than a standardized potency classification. Second, an exploratory meta-regression treating dose as a continuous moderator was performed only for DAI, given its consistent reporting across studies. Dose-category subgroup analysis was not performed for body weight because the available comparisons were unevenly distributed across dosing ranges and the dataset was limited to three studies.

### 2.6. Quality Assessment

Risk of bias was assessed using the Systematic Review Centre for Laboratory Animal Experimentation (SYRCLE) tool for animal studies [22] by two independent reviewers (C.R.M.-d.-O.-S. and S.R.F.-S.). Each domain was judged independently, with justification documented. Extraction procedures were designed to ensure that each dataset represented a unique experiment, avoiding duplication from overlapping publications. The certainty of evidence was evaluated using the preclinical adaptation of the GRADE framework by Hooijmans et al. [23], which integrates risk of bias (SYRCLE), inconsistency, indirectness related to clinical translation, imprecision, and potential publication bias. Each domain was assessed independently by C.R.M.-d.-O.-S. and -B.B.V., and the overall certainty for each outcome was rated as high, moderate, low, or very low. Formal funnel-plot asymmetry testing was not performed because fewer than ten studies were available per comparison; however, potential publication bias was assessed qualitatively, considering the limited evidence of preregistration and prospective protocol reporting in the included animal studies, as well as field-specific reporting practices and the possibility of small-study effects.

### 2.7. Sensitivity Analysis

Sensitivity analyses for DAI included leave-one-out recalculations at the study and dose-arm levels and influence diagnostics (externally studentized residuals, DFFITS, Cook’s distance, τ^2^-deletion, QE-deletion, and precision weights). Dose meta-regression (aqueous subset) and influence analyses were performed only for DAI, given its consistent reporting across studies.

### 2.8. Statistical Analysis

Outcomes were synthesized quantitatively when at least three studies reported comparable data. If fewer studies were available, findings were summarized narratively. Mechanistic add-on experiments (e.g., receptor agonists/antagonists or alternative models) were extracted qualitatively and synthesized narratively; only standard DSS-only vs. DSS + GQD comparisons were included in the quantitative meta-analysis. Standard mean differences (SMD) and mean differences (MD) were pooled with 95% confidence intervals (95% CI). We used the restricted maximum likelihood (REML) random-effects model adjusted with the Hartung–Knapp–Sidik–Jonkman method. Heterogeneity was quantified using the I^2^ statistic and its 95% CI [24]. Heterogeneity was considered substantial if I^2^ > 50%. Primary meta-analyses and forest plots were generated using RevMan Web v5.4.1 [25]. R v4.3.0 was utilized for advanced statistical procedures, including multilevel modeling for non-independent data, meta-regression, and comprehensive influence diagnostics [26]. To address shared-control multi-arm designs, we implemented multilevel models and performed arm-level influence diagnostics, explicitly accounting for non-independence and minimizing unit-of-analysis errors.

## 3. Results

### 3.1. Study Selection and Baseline Characteristics

**Figure 1:**
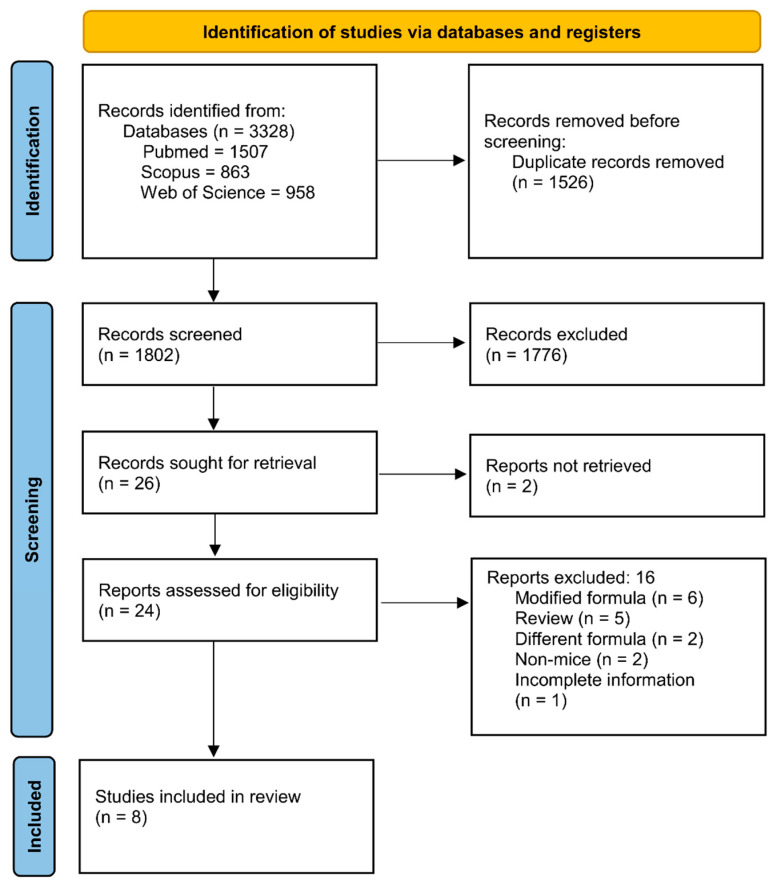
PRISMA 2020 flow diagram of study screening and selection.

A total of 3328 records were identified from electronic databases, including PubMed (n = 1507), Scopus (n = 863), and Web of Science (n = 958). After removing 1526 duplicate records, 1802 records remained for title and abstract screening. Of these, 1776 were excluded as irrelevant. Twenty-six reports were sought for retrieval, of which 2 could not be retrieved. The remaining 24 reports were assessed for eligibility via full-text review. Sixteen reports were excluded for the following reasons: modified formula (n = 6), review article (n = 5), different formula (n = 2), non-mice studies (n = 2), and incomplete information (n = 1). Reasons for full-text exclusions are summarized in Supplementary Table S5. Ultimately, 8 studies met the inclusion criteria and were included in the review (Figure 1).

The eight included studies comprised 209 mice (DSS-only controls: n = 60; GQD-treated: n = 149); 62.5% evaluated aqueous decoctions (5/8) and 37.5% evaluated ethanolic extracts (3/8; prepared with 75% ethanol or following the 75% ethanol protocol). All studies reported sex (7 male; 1 female). Most experiments used C57BL/6 mice, with ICR used only in one study. DSS protocols ranged from 2.5% to 3%, administered for 6–7 days (Table 1).

### 3.2. Intervention Characterization

Gegen Qinlian Decoction is a classical herbal formula traditionally composed of four ingredients: *Pueraria lobata*, *Scutellaria baicalensis*, *Coptis chinensis*, and *Glycyrrhiza uralensis* [8]. While the classical *Shang Han Lun* ratio is 8:3:3:2 (used by Yang et al. [28]), the majority of included studies (both aqueous and ethanolic) utilized a modified crude weight ratio of 5:3:3:2 (e.g., 15 g:9 g:9 g:6 g). Despite this variation in raw material proportion, reporting of downstream intervention characterization varied by extraction method and study design. Ethanol-based studies consistently reported HPLC-based fingerprinting and marker identification, with quantification more frequently available as numeric values in figures/tables. Among aqueous decoction studies, characterization approaches were diverse. Some studies relied on references to prior protocols. In contrast, recent high-quality investigations have utilized advanced metabolomic profiling to rigorously validate the aqueous matrix. Notably, Wang et al. [10] utilized HPLC-MS to quantify seven core bioactive markers (including puerarin, baicalin, and berberine), while Yang et al. [28] applied UPLC-Q-Orbitrap MS multi-omics analysis, identifying 98 distinct chemical constituents within the decoction. Regarding contamination control, explicit statements of endotoxin testing (e.g., LAL testing) were not consistently reported throughout the included literature. The reporting quality of the included studies was evaluated using the Reporting Characteristics Score (RCS-14), showing marked variability in the disclosure of herbal authentication, chemical quality control, and technical administration details (Table 2) [19]. Overall scores ranged from 8/14 to 12/14. Detailed quality control and chemical characterization of GQD preparations across the included studies are summarized in Supplementary Table S2.

### 3.3. Risk of Bias Assessment

**Figure 2:**
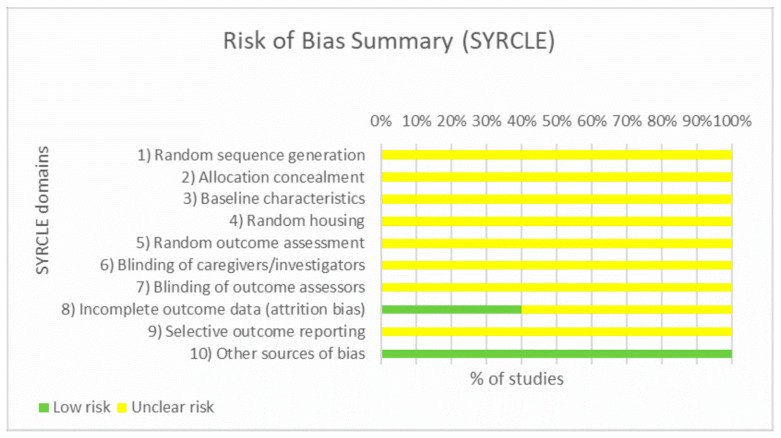
Summary of risk of bias across SYRCLE domains for the eight included studies.

The methodological quality of the eight included studies was assessed using the SYRCLE risk-of-bias tool [22]. Overall, methodological quality was limited due to insufficient reporting, a common characteristic of preclinical models. As summarized in Figure 2, the majority of domains were rated as unclear risk of bias, primarily due to the lack of explicit details regarding random sequence generation, allocation concealment, and blinding of caregivers or outcome assessors. Incomplete outcome data (attrition bias) presented a mixed profile: while approximately 40% of studies demonstrated low risk, the remainder were rated as unclear due to insufficient reporting of animal exclusions or withdrawals (e.g., lack of explanation for differences between starting n and analyzed n). Regarding selective reporting, although no obvious signs of bias were detected, all studies were rated as unclear due to the absence of pre-registered protocols. Finally, all studies were judged to have a low risk of “other bias,” as no additional sources of confounding were identified.

### 3.4. Quantitative Synthesis

#### 3.4.1. Disease Activity Index (DAI)

**Figure 3:**
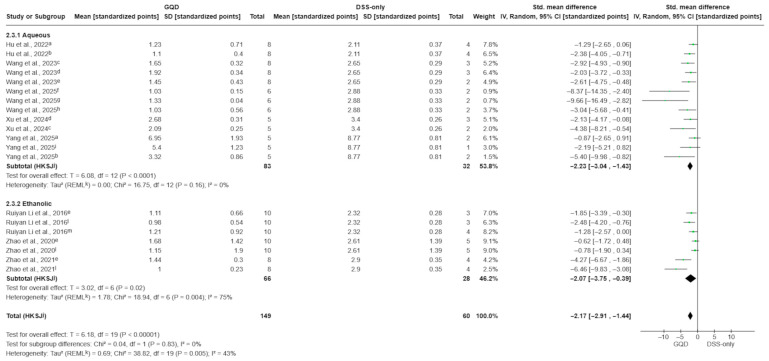
Effect of GQD on Disease Activity Index (DAI), stratified by extraction matrix (aqueous decoction vs. ethanolic extract). Both preparation types reduced DAI compared with DSS-only controls, with no significant subgroup difference (*p* = 0.83). Individual comparisons correspond to different GQD dose arms within each extract type [10,27,28,29,30,31,32,33]. Green squares indicate individual comparison effect sizes, with horizontal lines representing 95% confidence intervals; the black diamond denotes the pooled effect estimate. Footnotes: a = 5.85 g/kg; b = 17.55 g/kg; c = 5 g/kg; d = 2.5 g/kg; e = 7.5 g/kg; f = 4 g/kg; g = 16 g/kg; h = 8 g/kg; i = 11.7 g/kg; l = 1.5 g/kg; m = 0.3 g/kg; j = CI calculated using the Hartung–Knapp–Sidik–Jonkman method; k = τ^2^ calculated using the restricted maximum-likelihood method.

The effect of GQD on the DAI was evaluated across 20 comparisons from all included studies. Because several studies reported multiple GQD dose arms sharing a single DSS-only control, effect sizes were extracted per dose arm and pooled within extract-type subgroups (aqueous decoction vs. ethanolic extract). There was a significant reduction in DAI favoring GQD over DSS-only controls (SMD −2.17; 95% CI −2.91 to −1.44; *p* < 0.00001; I^2^ = 43%; Figure 3).

In subgroup analyses, both extract types significantly reduced DAI compared to DSS-only controls. Aqueous decoctions demonstrated a robust effect with no observed heterogeneity (SMD −2.23; 95% CI −3.04 to −1.43; *p* < 0.00001; I^2^ = 0%), while ethanolic extracts showed a similar magnitude of effect, but with substantial heterogeneity (SMD −2.07; 95% CI −3.75 to −0.39; *p* = 0.02; I^2^ = 75%). There were no significant differences between the extraction methods (test for subgroup differences: *p* = 0.83).

#### 3.4.2. Colon Length

**Figure 4:**
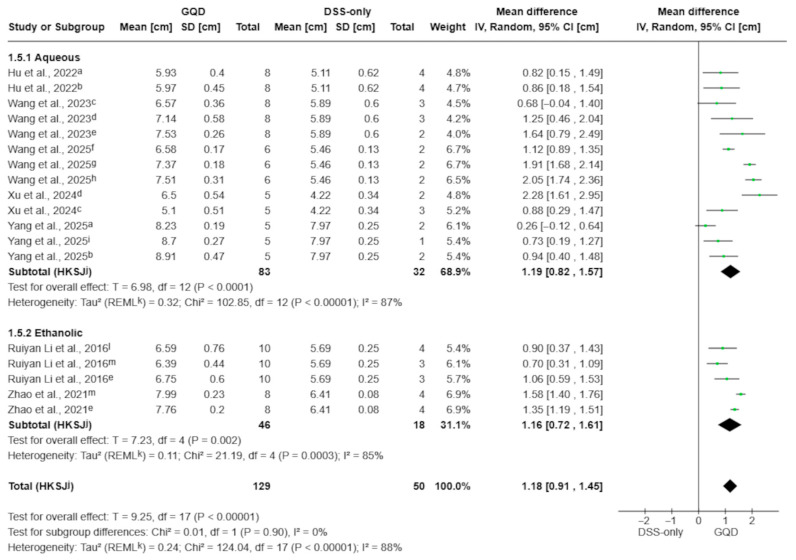
Effect of GQD on colon length, stratified by extraction matrix (aqueous decoction vs. ethanolic extract). GQD significantly attenuated DSS-induced colon shortening in both preparation types, with no significant subgroup difference (*p* = 0.90). Individual comparisons correspond to different GQD dose arms within each extract type [10,27,28,29,30,31,33]. Green squares indicate individual comparison effect sizes, with horizontal lines representing 95% confidence intervals; the black diamond denotes the pooled effect estimate. Footnotes: a = 5.85 g/kg; b = 17.55 g/kg; c = 2.5 g/kg; d = 5 g/kg; e = 7.5 g/kg; f = 4 g/kg; g = 16 g/kg; h = 8 g/kg; i = 11.7 g/kg; l = 0.3 g/kg; m = 1.5 g/kg; j = CI calculated using the Hartung–Knapp–Sidik–Jonkman method; k = τ^2^ calculated using the restricted maximum-likelihood method.

In the analysis of 18 comparisons from seven studies, GQD significantly prevented DSS-induced colon shortening (MD 1.18 cm; 95% CI 0.91 to 1.45; *p* < 0.00001; I^2^ = 88%; Figure 4). In subgroup analyses, there was comparable protection for both aqueous (MD 1.19 cm; 95% CI 0.82 to 1.57; *p* < 0.0001; I^2^ = 87%) and ethanolic extracts (MD 1.16 cm; 95% CI 0.72 to 1.61; *p* = 0.002; I^2^ = 85%). The difference between subgroups was not statistically significant (test for subgroup differences: *p* = 0.90), indicating that both preparation methods similarly attenuated colon shortening.

#### 3.4.3. Histological Score

**Figure 5:**
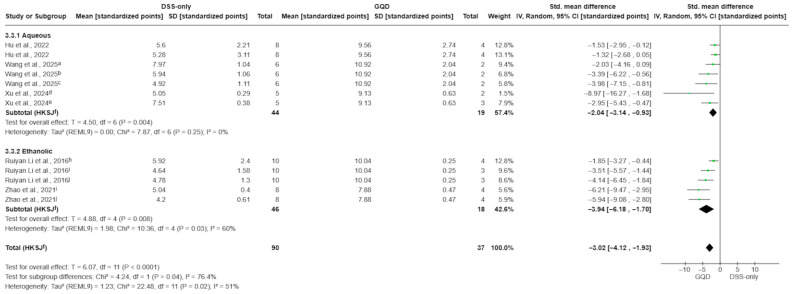
Effect of GQD on histological injury score, stratified by extraction matrix (aqueous decoction vs. ethanolic extract). Both preparation types improved histological injury compared with DSS-only controls; ethanolic extracts showed a larger pooled effect than aqueous decoctions (test for subgroup differences: *p* = 0.04). Individual comparisons correspond to different GQD dose arms within each extract type [27,29,30,31,33]. Green squares indicate individual comparison effect sizes, with horizontal lines representing 95% confidence intervals; the black diamond denotes the pooled effect estimate. Footnotes: a = 5.85 g/kg; b = 17.55 g/kg; c = 4 g/kg; d = 8 g/kg; e = 16 g/kg; f = 5 g/kg; g = 2.5 g/kg; j = 0.3 g/kg; h = CI calculated using the Hartung–Knapp–Sidik–Jonkman method; i = τ^2^ calculated using the restricted maximum-likelihood method.

Based on 12 treatment–control comparisons from five preclinical studies, GQD produced a large and statistically significant improvement in histological injury compared with DSS-only controls (SMD −3.02; 95% CI −4.12 to −1.93; *p* < 0.0001; I^2^ = 51%; Figure 5). In subgroup analyses, both extract types significantly reduced histological damage. Aqueous decoctions yielded an SMD of −2.04 (95% CI −3.14 to −0.93; *p* = 0.004; I^2^ = 0%), whereas ethanolic extracts yielded a larger effect size with an SMD of −3.94 (95% CI −6.18 to −1.70; *p* = 0.008; I^2^ = 60%). The test for subgroup differences was statistically significant (*p* = 0.04), indicating that ethanolic preparations demonstrated a stronger reduction in histological injury scores compared to aqueous decoctions in this analysis. This larger effect size should be interpreted cautiously given the substantially higher heterogeneity observed in ethanolic studies.

#### 3.4.4. Body Weight (Aqueous Decoctions)

Body weight outcomes were reported in seven treatment–control comparisons from three studies using aqueous GQD preparations [10,27,30]. Body weight was measured at the end of the study period, following the completion of DSS exposure and GQD treatment. For quantitative synthesis, body weight was analyzed as percent change from baseline, converting absolute values when baseline data were available; effects are reported as mean differences (percentage points).

**Figure 6:**
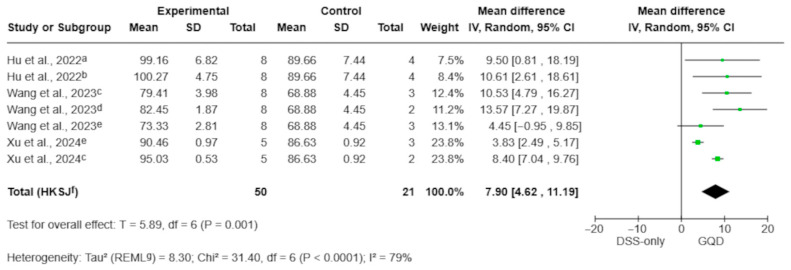
Effect of aqueous GQD preparations on body weight change (percentage points). Aqueous GQD mitigated DSS-induced body weight loss compared with DSS-only controls. Individual comparisons correspond to different GQD dose arms [10,27,30]. Green squares indicate individual comparison effect sizes, with horizontal lines representing 95% confidence intervals; the black diamond denotes the pooled effect estimate. No dose-category subgrouping was performed for this outcome. Footnotes: a = 5.85 g/kg; b = 17.55 g/kg; c = 5 g/kg; d = 7.5 g/kg; e = 2.5 g/kg; f = CI calculated using the Hartung–Knapp–Sidik–Jonkman method; g = τ^2^ calculated using the restricted maximum-likelihood method.

The pooled analysis showed that GQD significantly attenuated DSS-induced weight loss, with treated animals exhibiting greater preservation of body weight compared with DSS-only controls (MD 7.90 percentage points; 95% CI 4.62 to 11.19; *p* = 0.001; I^2^ = 79%; Figure 6). Dose-category subgroup analysis was not performed for body weight because the available comparisons were unevenly distributed across dosing ranges and the dataset was limited to three studies.

#### 3.4.5. Cytokines (Aqueous Decoctions)

**Figure 7:**
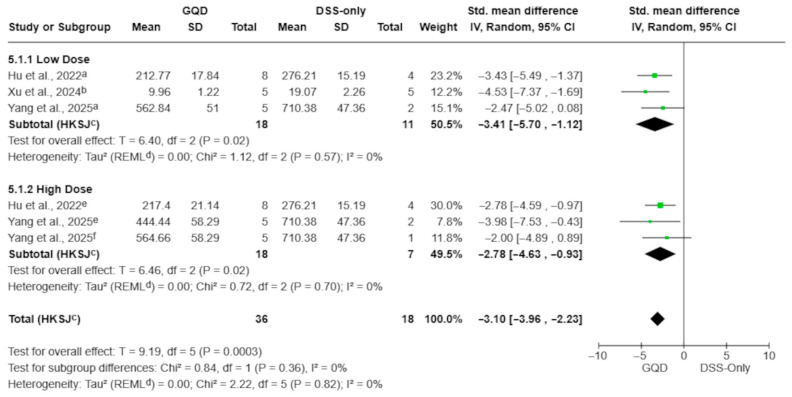
Effect of aqueous GQD preparations on circulating TNF-α levels, stratified by dose category (<7.5 g/kg vs. ≥7.5 g/kg). Dose categories are based on reported values (g/kg) without conversion to a unified raw-material–equivalent scale; therefore, this stratification should be interpreted as exploratory. Aqueous GQD reduced TNF-α compared with DSS-only controls. Individual comparisons correspond to different GQD dose arms [27,28,30]. Green squares indicate individual comparison effect sizes, with horizontal lines representing 95% confidence intervals; the black diamond denotes the pooled effect estimate. Footnotes: a = 5.85 g/kg; b = 17.55 g/kg; c = 5 g/kg; d = 11.7 g/kg; e = CI calculated using the Hartung–Knapp–Sidik–Jonkman method; f = τ^2^ calculated using the restricted maximum-likelihood method.

**Figure 8:**
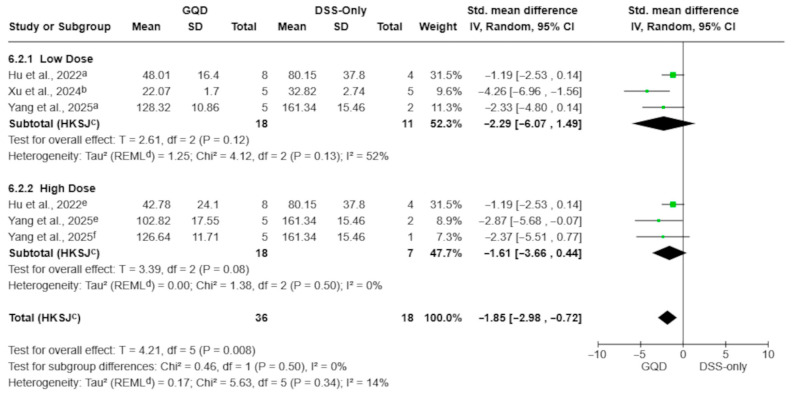
Effect of aqueous GQD preparations on circulating IL-6 levels, stratified by dose category (<7.5 g/kg vs. ≥7.5 g/kg). Dose categories are based on reported values (g/kg) without conversion to a unified raw-material–equivalent scale; therefore, this stratification should be interpreted as exploratory. Individual comparisons correspond to different GQD dose arms [27,28,30]. Green squares indicate individual comparison effect sizes, with horizontal lines representing 95% confidence intervals; the black diamond denotes the pooled effect estimate. Footnotes: a = 5.85 g/kg; b = 5 g/kg; e = 17.55 g/kg; f = 11.7 g/kg; c = CI calculated using the Hartung–Knapp–Sidik–Jonkman method; d = τ^2^ calculated using the restricted maximum-likelihood method.

**Figure 9:**
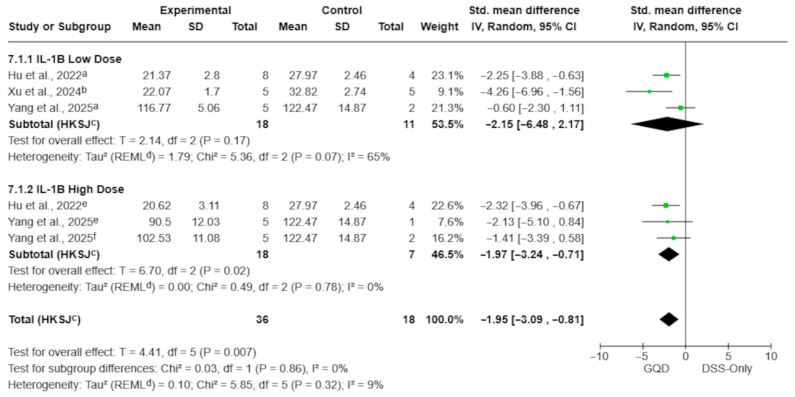
Effect of aqueous GQD preparations on circulating IL-1β levels, stratified by dose category (<7.5 g/kg vs. ≥7.5 g/kg). Dose categories are based on reported values (g/kg) without conversion to a unified raw-material–equivalent scale; therefore, this stratification should be interpreted as exploratory. Aqueous GQD reduced IL-1β compared with DSS-only controls. Individual comparisons correspond to different GQD dose arms [27,28,30]. Green squares indicate individual comparison effect sizes, with horizontal lines representing 95% confidence intervals; the black diamond denotes the pooled effect estimate. Footnotes: a = 5.85 g/kg; b = 5 g/kg; e = 17.55 g/kg; f = 11.7 g/kg; c = CI calculated using the Hartung–Knapp–Sidik–Jonkman method; d = τ^2^ calculated using the restricted maximum-likelihood method.

Aqueous GQD preparations significantly reduced circulating TNF-α, IL-6, and IL-1β levels compared with DSS-only controls (Figure 7, Figure 8 and Figure 9). The pooled effect for TNF-α showed a marked reduction (SMD −3.10; 95% CI −3.96 to −2.24; *p* = 0.0002; I^2^ = 0%; Figure 7), with similar reductions observed for IL-6 (SMD −1.85; 95% CI −2.98 to −0.72; *p* = 0.008; I^2^ = 14%; Figure 8) and IL-1β (SMD −1.95; 95% CI −3.09 to −0.81; *p* = 0.007; I^2^ = 9%; Figure 9).

Because cytokine outcomes were more consistently reported in aqueous preparations, dose-category analyses were restricted to this subset. When multiple GQD dose arms shared a single DSS-only control, effect sizes were extracted per dose arm and pooled within predefined dosing ranges (<7.5 g/kg vs. ≥7.5 g/kg), as described in the Methods.

For TNF-α, both dosing strata showed significant reductions: low-dose SMD −3.41 (95% CI −5.70 to −1.12; *p* = 0.02; I^2^ = 0%) and high-dose SMD −2.78 (95% CI −4.63 to −0.93; *p* = 0.02; I^2^ = 0%), with no evidence of subgroup differences (*p* = 0.36; Figure 7).

For IL-6, subgroup estimates did not reach statistical significance despite a consistent direction of effect. Low-dose extracts yielded an SMD of −2.29 (95% CI −6.07 to 1.49; *p* = 0.12; I^2^ = 52%), while high-dose extracts yielded an SMD of −1.61 (95% CI −3.66 to 0.44; *p* = 0.08; I^2^ = 0%); the subgroup difference test was not significant (*p* = 0.50; Figure 8).

For IL-1β, high-dose aqueous preparations showed a significant reduction (SMD −1.97; 95% CI −3.24 to −0.71; *p* = 0.02; I^2^ = 0%), whereas the low-dose subgroup did not (SMD −2.15; 95% CI −6.48 to 2.17; *p* = 0.17; I^2^ = 65%). However, no significant subgroup differences were detected (*p* = 0.86; Figure 9).

Finally, IL-10 was explored descriptively. Although individual aqueous study arms reported numerical increases relative to controls [27,30], the pooled estimate based on limited data (k = 3 comparisons) was not statistically significant (SMD 2.79; 95% CI −1.54 to 7.13; *p* = 0.11) and showed substantial heterogeneity (I^2^ = 64%), precluding definitive conclusions.

#### 3.4.6. Gut Microbiota Diversity (Shannon Index)

**Figure 10:**
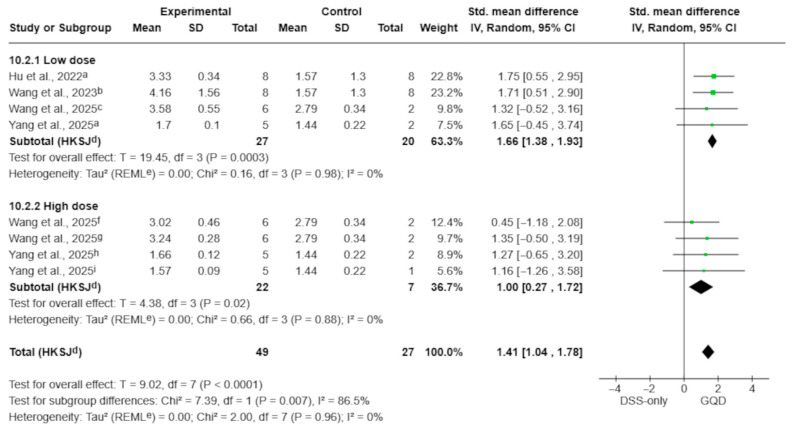
Effect of aqueous GQD preparations on gut microbiota diversity (Shannon index), stratified by dose category (<7.5 g/kg vs. ≥7.5 g/kg). Dose categories are based on reported values (g/kg) without conversion to a unified raw-material–equivalent scale; therefore, this stratification should be interpreted as exploratory. Both dose subgroups showed increased Shannon diversity compared with DSS-only controls; the between-subgroup difference was statistically significant (*p* = 0.007) [10,28,29,30]. Green squares indicate individual comparison effect sizes, with horizontal lines representing 95% confidence intervals; the black diamond denotes the pooled effect estimate. Footnotes: a = 5.85 g/kg; b = 5 g/kg; c = 4 g/kg; f = 8 g/kg; g = 16 g/kg; h = 17.55 g/kg; i = 11.7 g/kg; d = CI calculated using the Hartung–Knapp–Sidik–Jonkman method; e = τ^2^ calculated using the restricted maximum-likelihood method.

Based on eight treatment–control comparisons from four aqueous-extract studies, Gegen Qinlian Decoction (GQD) showed a consistent restorative effect on microbial diversity compared with DSS-only animals (SMD 1.41; 95% CI 1.04 to 1.78; *p* < 0.0001; I^2^ = 0%; Figure 10). Dose-stratified analyses demonstrated that both low-dose and high-dose aqueous decoctions improved Shannon Index values. However, the magnitude of the effect was larger in the low-dose subgroup (SMD 1.66; 95% CI 1.38 to 1.93; *p* = 0.0003; I^2^ = 0%) than in the high-dose subgroup (SMD 1.00; 95% CI 0.27 to 1.72; *p* = 0.02; I^2^ = 0%). The test for subgroup differences confirmed a statistically significant disparity (*p* = 0.007), with substantial heterogeneity between subgroup estimates (I^2^ = 86.5%), indicating dose-category variation in the magnitude of the effect.

#### 3.4.7. Additional Inflammatory and Barrier-Function Findings

Additional endpoints are summarized narratively below.

Two ethanolic extract studies [31,33] showed attenuation of DSS-induced weight loss: GQD-treated mice maintained higher final weights [33] or exhibited smaller percentage declines relative to baseline [31]. Although these results parallel the macroscopic protection observed in aqueous studies, only aqueous preparations displayed sufficient methodological consistency to support quantitative synthesis for body weight.

There were consistent decreases in colonic tissue TNF-α following ethanolic GQD extract administration. Ruiyan Li et al. [31] documented dose-dependent reductions across low- and high-dose groups by days 10–12, whereas Zhao et al. [33] reported marked decreases across dose levels, whether quantified as protein concentration or mRNA expression. Despite methodological variation, the direction of effect was uniform. Consistent with the aqueous subgroup, both preparation types showed convergent anti-TNF-α activity.

Both aqueous and ethanolic extracts consistently reduced colonic MPO activity, indicating attenuation of neutrophil-driven inflammation. Among aqueous studies [29,30], all reported doses produced lower MPO activity relative to DSS-only controls. Ethanolic extract studies [31,33] showed similarly consistent decreases across low- and high-dose arms. No preparation demonstrated a clear advantage over the other. Throughout all experiments, GQD consistently attenuated DSS-induced MPO elevation.

Aqueous GQD improved intestinal barrier function in DSS colitis by restoring tight-junction proteins (occludin and ZO-1 were upregulated) and junctional architecture, as shown by Western blot and confirmed by immunohistochemistry (IHC) and immunofluorescence (IF) [10,29,30]. Hu et al. [30] further reported broader barrier repair, with E-cadherin upregulated, claudin-2 downregulated, and recovery of the mucus layer, including MUC2 upregulation and increased goblet cells on Alcian blue/Periodic acid–Schiff (AB/PAS) staining [29,30].

Ethanolic studies provide complementary evidence consistent with mucosal regeneration and attenuation of inflammatory and oxidative signaling [31,32,33].

In aqueous studies, mechanistic endpoints included reduced lactate levels/production and inhibition of histone lactylation at specific sites (H3K18la, H3K23la) [27], as well as modulation of ferroptosis-related pathway proteins in colonic tissue (e.g., GPX4, SLC7A11) [34].

### 3.5. Sensitivity and Dose–Response Analyses (DAI)

In leave-one-out (LOO) sensitivity analyses for the main outcome, omitting any single study did not materially change the pooled effect estimate (Supplementary Figure S1). To account for multi-arm designs, we additionally conducted a treatment-arm–level influence analysis, sequentially removing each dosing arm while retaining the remaining arms from the same study; results remained stable across omitted arms (Supplementary Figure S2). Influence diagnostics (Cook’s distance and DFFITS) did not identify any influential outliers (Supplementary Figure S3).

In the continuous dose–response meta-regression, the estimated slope was negative but did not reach statistical significance (β = −0.144; *p* = 0.066; Supplementary Figure S4). Although this indicates a marginal, non-significant trend toward larger (more negative) effects at higher doses, dose explained a negligible proportion of between-study heterogeneity (R^2^ ≈ 0%), supporting broadly consistent effects across the evaluated dosing range (2.5–17.55 g/kg).

### 3.6. Certainty of Evidence

Using the preclinical adaptation of the GRADE framework [23], the overall certainty of evidence was rated as low for the primary outcome (DAI) and key secondary outcomes (colon length and histological score). The certainty was consistently downgraded across all outcomes due to risk of bias (insufficient reporting of randomization and blinding across SYRCLE domains), indirectness (limitations in translatability from murine DSS models to human clinical pathology), and suspected publication bias (limited evidence of preregistration and selective reporting safeguards in the primary preclinical literature, together with small-study effects that cannot be excluded). For immunological endpoints, certainty was additionally reduced because endotoxin/LPS testing was rarely reported, which may increase variability and could attenuate observed cytokine reductions (bias toward the null). Additionally, the certainty for colon length was further impacted by inconsistency arising from high heterogeneity (I^2^ = 88%). Detailed GRADE evidence profiles and specific reasons for downgrading are provided in Supplementary Table S4, and the summary of findings is presented in Table 3.

## 4. Discussion

### 4.1. Overview of Main Findings

The present systematic review and meta-analysis provides a consolidated evaluation of the pharmacological efficacy of Gegen Qinlian Decoction (GQD) in DSS-induced ulcerative colitis. Throughout the included preclinical studies, GQD consistently attenuated intestinal inflammation and improved clinical, macroscopic, and histological outcomes. Notably, aqueous preparations demonstrated exceptional reproducibility, with essentially absent heterogeneity across key outcomes, despite the inherent biological variability of the DSS model. This observation is particularly striking given that DSS-induced injury is highly sensitive to host genetics, microbiota composition, and experimental conditions, all of which can substantially influence disease severity [35].

Beyond confirming efficacy, our findings identify the extraction matrix as a critical determinant of pharmacological consistency. While both aqueous and ethanolic extracts showed biological activity, the traditional aqueous decoction consistently yielded more stable and reproducible effects. Dose-category and meta-regression analyses did not provide consistent evidence of incremental benefit at higher doses across the evaluated range. Overall, these results suggest that the aqueous formulation represents a pharmacologically consistent preparation and is best suited for continued preclinical investigation and translational consideration.

### 4.2. Therapeutic Efficacy: Clinical Signs and Inflammatory Control

GQD treatment produced a pronounced reduction in disease activity index (DAI), reflecting improvements in stool consistency and rectal bleeding, alongside preservation of body weight [10,27,28,29,30,31,32,33]. In acute DSS colitis, weight loss is rapid and largely driven by systemic inflammatory burden; therefore, attenuation of this decline suggests that GQD exerts effects extending beyond local mucosal protection [10,27,30]. These clinical benefits were paralleled by macroscopic preservation of colon length, an outcome that integrates epithelial injury, edema, and smooth muscle contraction [34]. At the microscopic level, GQD substantially reduced histological damage, preserving crypt architecture and limiting inflammatory infiltration [27,29,30,31,33].

The convergence of clinical, macroscopic, and histological improvements supports the interpretation that GQD modulates core pathogenic processes rather than merely alleviating symptoms. These phenotypic effects are consistent with suppression of key inflammatory mediators. Pooled analyses demonstrated significant reductions in TNF-α and IL-6, cytokines that orchestrate the acute inflammatory cascade in DSS-induced injury [10,29,30,31,33]. Mechanistic studies provide a coherent framework for these observations. Suppression of TLR4/NF-κB signaling has been shown to reduce pro-inflammatory cytokine production [31], while inhibition of the IL-6/JAK2/STAT3 axis limits Th17 expansion and promotes restoration of the Th17/Treg balance [33]. Collectively, these findings indicate that the cytokine reductions observed in our analysis likely reflect coordinated modulation of upstream inflammatory pathways rather than isolated downstream effects [31,33].

### 4.3. Extraction Matrix and Supramolecular Consistency

A central finding of this synthesis is the striking contrast in reproducibility between extraction methods. While ethanolic extracts consistently produced biological effects, their magnitude varied substantially between studies. All included ethanolic studies reported using 75% ethanol; therefore, differences in ethanol percentage are unlikely to be a primary driver of heterogeneity within this subgroup. This suggests other sources of variability (e.g., herb sourcing, herb-to-solvent ratio, extraction time/temperature, evaporation/reconstitution conditions, and model- and study-level differences), which were not consistently reported across studies. In contrast, aqueous decoctions demonstrated tightly clustered effects with minimal heterogeneity. However, very low statistical heterogeneity (e.g., I^2^ ≈ 0%) in a small preclinical dataset should be interpreted cautiously. With few studies, I^2^ has limited power to detect between-study variability, and apparent homogeneity may partly reflect shared DSS protocols and outcome assessment practices. Thus, these estimates are best interpreted as consistency within the available experimental context rather than evidence of uniform effects across broader settings. Even acknowledging these constraints, the observed contrast between matrices, greater variability in ethanolic extracts versus more tightly clustered effects in aqueous decoctions, supports the hypothesis that extraction-dependent differences in the physicochemical organization of the formula may contribute to reproducibility. Traditional boiling may facilitate supramolecular organization in aqueous decoctions. Experimental evidence has shown that alkaloids such as berberine can form ionic complexes with flavonoids including baicalin and wogonoside, resulting in structures with enhanced lipophilicity and absorption compared to isolated compounds [36]. However, the studies did not report direct physicochemical characterization (e.g., DLS, TEM, or zeta potential) to confirm supramolecular/colloidal assemblies or decoction-derived nanoparticles; therefore, this mechanism is presented as a plausible rationale rather than a feature directly demonstrated within the included studies. More recently, naturally occurring protein–polysaccharide nanoparticles of approximately 110 nm have been identified in aqueous GQD preparations, acting as scaffolds that stabilize and transport multiple bioactive constituents through the gastrointestinal tract [37]. Additional micro- and nano-aggregates have also been shown to facilitate trans-epithelial transport of baicalin and increase antioxidant activity in vitro [38]. By comparison, ethanolic extraction may reduce many hydrophilic macromolecules required for formation of these assemblies, resulting in looser mixtures that are more susceptible to batch-to-batch variation. This mechanistic framework provides a plausible explanation for the superior reproducibility observed with aqueous preparations and supports the concept that preservation of supramolecular architecture may be an important contributor to pharmacological consistency. Importantly, these endogenous supramolecular/nanoparticle assemblies described in aqueous decoctions differ from engineered nanoparticle-enhanced formulations, which were excluded by design.

Interestingly, our RCS-14 assessment suggests that ethanolic studies more frequently reported small-molecule chemical QC (e.g., marker quantification), yet their in vivo effects were less reproducible. This implies that marker-based QC alone may not capture matrix-level features that influence delivery and bioactivity. For example, recent work shows that aqueous GQD can form naturally occurring protein–polysaccharide nanoparticles (~110 nm) that encapsulate constituents and enhance colonic absorption, which would not be reflected by quantifying only a few markers [37]. In parallel, multi-omics profiling of a classic GQD identified a broad chemical space (98 constituents) linked to coordinated metabolic and microbiota shifts, again suggesting that a limited QC panel may miss higher-order “holistic” properties relevant to biological consistency [28].

### 4.4. Dose–Response and Translational Considerations

Overall, increasing the dose of aqueous GQD did not yield clearly proportional gains in efficacy across pooled outcomes. Studies employing lower doses (<7.5 g/kg) achieved improvements comparable to those using higher doses (≥7.5 g/kg). The apparent discrepancy between dose-dependent effects reported in individual ethanolic studies and the absence of a clear dose–response relationship in pooled aqueous analyses may reflect differences in analytical scale rather than a true pharmacological inconsistency. Dose-dependent trends observed within individual experiments represent intra-study pharmacodynamic responses under controlled conditions [31,33], whereas meta-regression evaluates whether dose systematically explains between-study variability at the population level.

In the pooled dataset, aqueous GQD preparations demonstrated robust therapeutic effects over a broad dosing range, with minimal heterogeneity and no consistent dose–response gradient [10,27,28,29,30], a pattern consistent with an early plateau of efficacy. Notably, the meta-regression showed a marginal, non-significant trend suggesting that higher doses might further reduce DAI (*p* = 0.066), but dose explained negligible heterogeneity (R^2^ ≈ 0%), supporting overall consistency across the tested range. In contrast, ethanolic extracts, analyzed primarily at the individual-study level, frequently reported dose-dependent molecular responses, particularly for inflammatory markers such as TNF-α [31,33]. This divergence is consistent with differences in extraction matrix and delivery efficiency, whereby aqueous preparations may achieve pharmacological saturation at lower doses due to supramolecular complex formation and nanoparticle-mediated delivery [35,36,37], while ethanolic extracts rely more strongly on concentration-dependent exposure to elicit comparable effects [31,33].

Importantly, these findings do not imply superior intrinsic potency of one preparation over the other, but rather highlight formulation-dependent differences in reproducibility, dose sensitivity, and translational robustness. These observations reinforce the translational relevance of doses aligned with traditional clinical practice and support the ethical principle of minimizing unnecessary exposure in preclinical research, consistent with the 3Rs framework [39].

### 4.5. Mechanisms of Action: Ecological and Homeostatic Regulation

The therapeutic effects of GQD appear to arise from complementary actions on the intestinal ecosystem and epithelial homeostasis. At the ecological level, aqueous GQD consistently restored microbial diversity disrupted by DSS exposure [10,28,29]. Several studies reported enrichment of taxa linked to barrier integrity (e.g., Akkermansia) and broader remodeling of Bacteroides abundance, changes associated with improved barrier function and metabolic balance [10,28,29]. Host–microbe metabolic cross-talk also appears central to these effects. In a multi-omics study, integrated network pharmacology, serum metabolomics, and 16S profiling identified prominent modulation of tryptophan metabolism, arginine/proline metabolism, and branched-chain amino acid biosynthesis, alongside increases in Firmicutes/Verrucomicrobia and (at the genus level) Akkermansia and Muribaculum [28]. Consistent with these shifts, GQD attenuated intrinsic apoptosis in colon tissue (↓Bax, ↓cytochrome c, ↓caspase-3; ↑Bcl-2), supporting a metabolite–microbiota axis linked to reduced epithelial injury [28].

Activation of epithelial PPAR-γ restricts luminal oxygen availability, limiting expansion of facultative anaerobic pathobionts such as Enterobacteriaceae [30]. Hu et al. (2022) reinforced this pathway using a PPAR-γ perturbation arm: pharmacologic antagonism with T0070907 attenuated the GQD-associated suppression of iNOS/nitrate signaling and the reduction in Enterobacteriaceae/*E. coli* and colonic LPS, supporting partial PPAR-γ dependence of the dysbiosis phenotype [30]. A second perturbation experiment implicated the AhR/IL-22 axis: in Wang et al. (2023) [10], mice received the AhR antagonist CH223191 prior to GQD (5 g/kg), and AhR blockade abrogated key barrier-restorative effects of GQD. CH223191 also reduced the GQD-associated increase in IL-22–producing ILC3 and downstream AhR signaling (e.g., CYP1A1), supporting involvement of the AhR/IL-22 axis [10].

Pathway-level analyses further suggest coordinated suppression of inflammatory and stress signaling. Wang et al. (2025) [29] integrated network pharmacology with in vivo validation, implicating p53/Akt/STAT3 and MAPK (p38/JNK/ERK) signaling and showing concordant reductions in pathway phosphorylation in colon tissue following GQD treatment. In the same study, GQD improved barrier-related proteins (ZO-1, Occludin, and MUC2), consistent with reduced intestinal permeability, and microbial remodeling was linked to host signaling through Spearman correlations (e.g., Lactobacillus/Allobaculum inversely associated with p-p53 and p-JNK, and Bacteroides/Romboutsia showing positive associations with p-p53, p-Akt, or p-JNK) [29].

Mechanistic evidence from ethanolic preparations is more limited but aligns with suppression of innate inflammatory signaling, oxidative stress, and immune polarization. In Ruiyan Li et al. (2016) [31], GQD attenuated DSS colitis alongside reduced colonic TLR4 expression and NF-κB activation, with concurrent improvements in oxidative/inflammatory readouts (e.g., lower MPO and MDA, higher GSH). Complementary LPS-stimulated RAW264.7 experiments showed reductions in pro-inflammatory mediators and NO/ROS generation, supporting a direct macrophage-modulatory component [31]. In a related study, restoration of the Th17/Treg balance was also reported, alongside suppression of IL-6/JAK2/STAT3 signaling, supported by quantification of Th17 and Treg populations (e.g., CD3 + IL-17A+ and CD25 + Foxp3+) in Peyer’s patches and spleen [33].

At the epithelial-program level, earlier work further suggested phase-dependent Notch dysregulation in DSS colitis and proposed that GQD restores mucosal homeostasis by normalizing this imbalance [32]. In acute colitis, GQD downregulated Hes1 and Notch-related proteins (RBP-J and MAML), increased secretory differentiation (Math1) and goblet cells (AB/PAS; MUC2), consistent with barrier restitution; whereas in chronic colitis, Notch markers shifted in the opposite direction with increased proliferative activity (Ki67), supporting context-dependent epithelial renewal programs [32].

Additional mechanistic layers reported in recent aqueous studies include site-specific suppression of histone lactylation (e.g., H3K18la and H3K23la), a process directly linked to inhibition of pro-inflammatory M1 macrophage polarization [27], and inhibition of ferroptosis-associated pathways [34]. In Xu et al. [27], exogenous lactate supplementation partially reversed the protective phenotype and counteracted the shift away from M1 (CD86+) toward M2 (CD163+) macrophage polarization, supporting functional involvement of the lactate–histone lactylation axis in DSS colitis. Together, these findings suggest that GQD acts less as a single-pathway inhibitor and more as a dynamic regulator of intestinal homeostasis, beyond a purely anti-inflammatory agent [10,27,30,32].

### 4.6. Contextualization of the Acute DSS Model and Timing of Administration

Although all included studies employed the acute DSS model, this framework remains highly relevant for studying active disease flares characterized by epithelial barrier disruption and robust innate immune activation. In C57BL/6 mice, early DSS exposure triggers infiltration of neutrophils and macrophages and a pronounced cytokine surge, whereas adaptive immune responses become prominent only later [40]. Thus, efficacy observed in acute models reflects interception of early pathogenic drivers that initiate disease progression [41,42]. It is also noteworthy that included studies employed different administration protocols. Some initiated GQD treatment concomitantly with DSS exposure, modeling co-treatment designs, while others administered GQD after colitis induction, reflecting therapeutic or recovery-oriented approaches. Importantly, GQD exerted beneficial effects under both paradigms, suggesting a dual pharmacodynamic profile encompassing prevention of the initial inflammatory cascade and promotion of mucosal repair. However, beyond biological timing differences, the included literature also showed variability in how the GQD intervention itself was described and chemically characterized, which may influence reproducibility and cross-study comparability.

### 4.7. Intervention Reporting Quality and Reproducibility Assessment (RCS-14)

To better interpret the reproducibility of the herbal intervention, we evaluated how completely GQD preparation and administration were reported across studies using the RCS-14 framework (Table 2), guided by PRISMA-CHM 2020 domains that emphasize intervention transparency and replicability [19]. Differences in RCS-14 scores should mainly be understood as variation in reporting detail, rather than as a direct indicator of experimental quality.

Across the included studies, key intervention parameters such as dose and treatment duration were usually reported, which supports the interpretation of pooled efficacy outcomes. However, some practical details that matter for pharmacological replication, such as explicit route terminology, vehicle specification, or numerical marker quantification, were not consistently stated. Following our strict evidence-direct approach, these elements were recorded as not reported (NR) when they were not explicitly documented, to avoid assuming details that were not clearly described (Supplementary Table S2).

Most studies included some form of chemical characterization, typically through chromatographic fingerprinting and marker identification. In contrast, numerical marker quantification was less consistently available and, in some cases, could only be inferred from graphical presentation. This matters because reporting quantitative marker content improves batch-to-batch comparability and makes it easier to interpret results across studies, particularly when extraction methods differ.

Overall, ethanolic extracts more often included explicit descriptions of chemical quality control and technical administration details, whereas aqueous decoctions occasionally provided these elements in a less detailed manner (Table 2; Supplementary Table S2). Interestingly, this pattern did not translate into greater consistency in effect sizes: aqueous preparations showed tightly clustered effects with minimal heterogeneity across major outcomes, while ethanolic extracts—despite broadly similar methods—displayed greater variability between studies. This suggests that reporting completeness and biological reproducibility do not always align, and it reinforces the extraction matrix as an important contributor to consistency, in line with evidence that traditional decoction may preserve supramolecular assemblies and naturally occurring macromolecular carriers. Taken together, these observations support future standardization efforts that strengthen intervention reporting while also preserving preparation features that may be relevant for reproducible efficacy.

This apparent divergence between marker-based QC reporting and biological reproducibility is discussed in Section 4.3, where we highlight that matrix-level properties may not be captured by routine marker panels.

### 4.8. Strengths and Limitations

This study represents the first systematic synthesis to stratify GQD efficacy by extraction solvent and to explore dose effects, providing insight into formulation-dependent reproducibility. By integrating quantitative outcomes with mechanistic interpretation, this analysis moves beyond confirmation of efficacy toward identification of physicochemical determinants that shape pharmacological performance. Several limitations warrant consideration. First, all included studies employed the acute DSS model, which does not fully recapitulate the relapsing immunopathology of human ulcerative colitis. Nevertheless, this model remains the gold standard for evaluating therapeutic efficacy during active disease flares [40,41,42]. Second, reporting of randomization and blinding was frequently unclear, a limitation common to preclinical research [22]. Accordingly, the SYRCLE assessment yielded frequent ‘unclear’ judgments in domains related to randomization and blinding, which lowers certainty and supports cautious interpretation of pooled effect sizes, even when statistical heterogeneity appears low. Third, explicit certification of endotoxin-free preparations or endotoxin/LPS testing (e.g., LAL assay) was rarely reported in the included studies, which limits interpretability for cytokine outcomes. Unmeasured LPS could increase baseline inflammatory tone and/or add variability; if present in the intervention preparation, it could partially counteract anti-inflammatory activity and attenuate observed cytokine reductions (bias toward the null). However, because endotoxin status was not systematically reported, the direction and magnitude of any bias cannot be determined. Fourth, the ethanolic-extract subgroup comprised only three studies, limiting the precision and power of between-solvent subgroup comparisons and restricting mechanistic inference for ethanolic preparations. Finally, the restriction to English-language databases may have excluded regionally indexed studies, reinforcing the focus on a globally accessible and verifiable evidence base.

## 5. Conclusions

In DSS-induced colitis models, Gegen Qinlian Decoction demonstrates robust therapeutic efficacy across clinical, macroscopic, and histological outcomes. Across studies, aqueous preparations showed the most consistent effects, whereas ethanolic extracts showed less consistent effect estimates across studies. These findings support the translational relevance of GQD across commonly used dosing ranges and highlight traditional aqueous decoction as a reproducible formulation for preclinical research. Future studies should prioritize standardized preparation workflows, improved chemical characterization, and mechanistic experiments designed to directly test formulation-dependent differences, thereby strengthening reproducibility and supporting more efficient clinical translation.

## Figures and Tables

**Table 1:** Baseline characteristics of included studies.

Study (Author, Year)	Mouse Strain	Sex	Age (Weeks)	Weight (g)	DSS Protocol	GQD Dose (g/kg, as Reported)	GQD Administration Schedule (Relative to DSS)	n/Group	Key Outcomes Reported	Extract Type
Wang et al., 2023 [10]	C57BL/6J	Male	6–8	20–22	3% DSS for 6 days	2.5, 5.0, 7.5	During DSS (days 1–6)	8	DAI, CL	Aqueous decoction
Xu et al., 2024 [27]	C57BL/6	Male	8	≈25	2.5% DSS for 7 days	2.5, 5.0	During DSS (days 1–10)	5	DAI, CL, HS	Aqueous decoction
Yang et al., 2025 [28]	ICR	Male	10–12	NR	3% DSS for 7 days	5.85, 11.7, 17.55	Post-DSS (days 8–21)	5	DAI, CL	Aqueous decoction
Wang et al., 2025 [29]	C57BL/6J	Male	4	18–22	3% DSS for 7 days	4, 8, 16	During DSS (days 1–7)	6	DAI, CL, HS	Aqueous decoction
Hu et al., 2022 [30]	C57BL/6J	Male	6–8	18–22	2.5% DSS for 7 days	5.85, 17.55	Post-DSS (days 8–14)	8	DAI, CL, HS	Aqueous decoction
Ruiyan Li et al., 2016 [31]	C57BL/6	Male	6–8	18–22	5% DSS for 7 days	0.3, 1.5, 7.5	During DSS (days 3–10)	10	DAI, CL, HS	Ethanolic extract (75%)
Zhao et al., 2020 [32]	C57BL/6	Female	6–8	18–22	3% DSS for 7 days	1.5, 7.5	During DSS (days 5–11)	10	DAI	Ethanolic extract (75%)
Zhao et al., 2021 [33]	C57BL/6	Female	8	18–22	3% DSS for 7 days	1.5, 7.5	During DSS (days 4–10)	8	DAI, CL, HS	Ethanolic extract (75%)

Notes: The technical route of administration was coded as “reported” only when explicitly stated as gavage or intragastric. If the study reported only “orally administered” or “given,” the technical route was coded as not specified (NR). Animal source/supplier information is reported in Supplementary Table S3. Dose reporting: Dose values are shown as reported (g/kg). When explicitly stated, we noted whether dosing referred to crude-drug equivalents or dried extract mass. Because extraction yields and dosing bases were inconsistently reported, doses were not converted to a unified raw-material–equivalent scale; thus, dose-tier analyses are exploratory rather than standardized potency comparisons. Abbreviations: DAI, Disease Activity Index; CL, colon length; HS, histological score; NR, not reported.

**Table 2:** Reporting quality assessment of included studies using the Reporting Characteristics Score (RCS-14).

Study (Ref.)	Extract Type	Ident. (/3)	Proc. (/4)	QC (/3)	Admin. (/4)	Total (/14)
Wang et al., 2023 [10]	Aqueous	1	4	2	3	10
Xu et al., 2024 [27]	Aqueous	1	4	0	4	9
Yang et al., 2025 [28]	Aqueous	2	4	2	3	11
Wang et al., 2025 [29]	Aqueous	1	4	0	4	9
Hu et al., 2022 [30]	Aqueous	1	4	2	3	10
Ruiyan Li et al., 2016 [31]	Ethanolic	1	4	3	4	12
Zhao et al., 2021 [33]	Ethanolic	1	4	2	4	11
Zhao et al., 2020 [32]	Ethanolic	1	4	3	4	12

Abbreviations: Ident., botanical identity and composition; Proc., extraction processing; QC, chemical quality control; Admin., pharmacological administration. Scoring rule: Items were scored only when explicitly reported in the main text, figures, or Supplementary Materials; otherwise, they were coded as not reported (NR). Detailed evidence supporting each item is provided in Supplementary Table S2.

**Table 3:** Summary of Findings: Gegen Qinlian Decoction (GQD) compared with vehicle control in DSS-induced colitis. Population: Mice with DSS-induced colitis. Intervention: GQD (aqueous decoction or ethanolic extract). Comparison: Vehicle/DSS-only control.

Outcome	No. of Studies	Relative Effect (95% CI)	Certainty of Evidence (Adapted GRADE)	Anticipated Effects
Disease Activity Index (DAI)	8	SMD −2.17 [−2.91, −1.44]	⨁⨁◯◯ LOW	GQD may reduce disease activity versus vehicle across aqueous and ethanolic preparations (low-certainty evidence).
Colon Length (cm)	7	MD 1.18 cm [0.91, 1.45]	⨁⨁◯◯ LOW	GQD may preserve colon length (≈1.2 cm longer) versus vehicle (low-certainty evidence).
Histological Score	5	SMD −3.02 [−4.12, −1.93]	⨁⨁◯◯ LOW	GQD may reduce histological tissue injury versus vehicle (low-certainty evidence).

Abbreviations: cm, centimeters; CI, confidence interval; DAI, disease activity index; SMD, standardized mean difference; MD, mean difference. Note: The analysis includes aqueous decoctions (5 studies) and ethanolic extracts (3 studies). Certainty of evidence was assessed using an adapted GRADE approach for preclinical animal studies (Hooijmans et al. [23]). Certainty of evidence (GRADE): ⊕⊕⊕⊕ = high; ⊕⊕⊕◯ = moderate; ⊕⊕◯◯ = low; ⊕◯◯◯ = very low. Heterogeneity was minimal in aqueous studies for DAI and higher in ethanolic extracts; certainty was downgraded mainly due to risk of bias and indirectness.

## Data Availability

No new data were created in this study.

## Supplementary Materials

The following supporting information can be downloaded at: https://www.mdpi.com/article/10.3390/ph19020277/s1, Table S1: Full Search Strategies (PubMed, Scopus, Web of Science). Table S2: Detailed Characterization and Audit Trail of Reporting Quality (RCS-14) for Included Studies. Table S3: Animal Source/supplier. Table S4: Detailed GRADE Evidence Profiles and Reasons for Downgrading. Table S5: Full-text excluded studies and reasons for exclusion. Figure S1: Leave-one-out sensitivity analysis (Study-level). Figure S2: Leave-one-out sensitivity analysis (Arm-level). Figure S3: Influence diagnostics (Cook’s distance, DFFITS). Figure S4: Meta-regression of Dose vs. DAI (Aqueous extracts).

## Abbreviations

The following abbreviations are used in this manuscript:

CL	Colon Length
CI	Confidence interval
DAI	Disease Activity Index
DSS	Dextran sulfate sodium
GQD	Gegen Qinlian Decoction
HK-SJ	Hartung–Knapp–Sidik–Jonkman
HS	Histological Score
IL	Interleukin
I^2^	Higgins’ I-squared statistic (heterogeneity)
MD	Mean difference
NF-κB	Nuclear factor kappa B
PPAR-γ	Peroxisome proliferator-activated receptor gamma
PRISMA	Preferred Reporting Items for Systematic Reviews and Meta-Analyses
SMD	Standardized mean difference
SYRCLE	Systematic Review Centre for Laboratory Animal Experimentation
TLR4	Toll-like receptor 4
UC	Ulcerative colitis
