# Supplementary material for: Extraction Matrix Shapes the Efficacy of Gegen Qinlian Decoction in DSS-Induced Colitis: A Preclinical Systematic Review and Meta-Analysis
Source: Pharmaceuticals (Basel). 2026 Feb 6;19(2):277. doi: 10.3390/ph19020277 (PMC12943224; doi:10.3390/ph19020277)
Supplement: Supplementary file 1 [file pharmaceuticals-19-00277-s001.zip › pharmaceuticals-4133969-supplementary.pdf]

Supplementary Materials

Extraction Matrix Shapes the Efficacy of Gegen Qinlian Decoction in DSS-Induced Colitis: A Preclinical Systematic Review and Meta-Analysis

Table of Contents

Supplementary Tables

- **Table S1.** Full Search Strategies (PubMed, Scopus, Web of Science).
- **Table S2.** Detailed Characterization and Audit Trail of Reporting Quality (RCS-14) for Included Studies.
- **Table S3.** Animal Source/supplier
- **Table S4.** Detailed GRADE Evidence Profiles and Reasons for Downgrading.
- **Table S5.** Full-text excluded studies and reasons for exclusion

Supplementary Figures

- **Figure S1.** Leave-one-out sensitivity analysis (Study-level).
- **Figure S2.** Leave-one-out sensitivity analysis (Arm-level).
- **Figure S3.** Influence diagnostics (Cook’s distance, DFFITS).
- **Figure S4.** Meta-regression of Dose vs. DAI (Aqueous extracts).

Supplementary Table S1. Full search strategies (July 2025).

Database	Search Query (Free-text)	Hits (n)
PubMed	("dextran sulfate sodium" OR DSS OR "dextran sodium sulfate") AND ("ulcerative colitis" OR colitis) AND ("Gegen Qinlian" OR "Gegen Qin Lian" OR GQD OR "Chinese herbal" OR "herbal medicine" OR phytotherapy OR decoction OR "Chinese medicine") AND ("animal model" OR "preclinical" OR mice OR mouse OR murine OR rodent OR "in vivo")	1,507
Scopus	("dextran sulfate sodium" OR DSS OR "dextran sodium sulfate") AND ("ulcerative colitis" OR colitis) AND ("Gegen Qinlian" OR "Gegen Qin Lian" OR GQD OR "Chinese herbal" OR "herbal medicine" OR phytotherapy OR decoction OR "Chinese medicine") AND ("animal model" OR "preclinical" OR mice OR mouse OR murine OR rodent OR "in vivo")	863
Web of Science	("dextran sulfate sodium" OR DSS OR "dextran sodium sulfate") AND ("ulcerative colitis" OR colitis) AND ("Gegen Qinlian" OR "Gegen Qin Lian" OR GQD OR "Chinese herbal" OR "herbal medicine" OR phytotherapy OR decoction OR "Chinese medicine") AND ("animal model" OR "preclinical" OR mice OR mouse OR murine OR rodent OR "in vivo")	958
**Total**		**3,328**

Note: Searches were conducted in July 2025 using free-text terms.

Supplementary Table S2. Detailed Reporting Evidence Audit (RCS-14)

Coding:

Y = explicitly reported in the article text, figures, or supplementary material.
NR = not reported.
Y (Ref) = reported by explicit reference to a prior publication describing a reproducible protocol (details not reprinted in the current manuscript).

Parameter (RCS-14)	Wang 2023	Xu 2024	Hu 2022	Yang 2025	Wang 2025	Li 2016	Zhao 2020	Zhao 2021
Preparation type	Aqueous	Aqueous	Aqueous	Aqueous	Aqueous	Ethanolic	Ethanolic	Ethanolic
Raw herbs / Ratio	Y (15:9:9:6)	Y (15:9:9:6)	Y (15:9:9:6)	Y (8:3:3:2)	Y (15:9:9:6)	Y (20:12:12:8)	Y (Ref [r])	Y (5:3:3:2)
Authentication	NR	NR	NR	Y (Ping Wang)	NR	NR	NR	NR
Voucher specimen	NR	NR	NR	NR	NR	NR	NR	NR
Solvent	Water (Y)	Water (Y)	Water (Y)	Water (Y)	Water (Y)	75% EtOH (Y)	Y (Ref [r])	75% EtOH (Y)
Extraction params	Y	Y	Y	Y	Y	Y	Y (Ref [r])	Y
Number of cycles	Y	Y	Y	Y	Y	Y	Y (Ref [r])	Y
Conc. / Drying	Y	Y	Y	Y	Y	Y	Y (Ref [r])	Y
Fingerprint method	Y (HPLC-MS)	NR	Y (HPLC-UV)	Y (LC-MS)	NR [o]	Y (HPLC-UV)	Y (HPLC-UV)	Y (HPLC-UV)
Markers identified	Y (7)	NR	Y (5)	Y (98)	NR [o]	Y (6)	Y (6)	Y (6)
Marker quant.	NR [l]	NR	NR [k]	NR [m]	NR	Y (%)	Y (% [t])	NR [u]
Dose (numeric)	Y	Y	Y	Y	Y	Y	Y	Y
Route (tech. term)	NR [h]	Y (Gavage)	NR [h]	NR [i]	Y (Intragastric)	Y (Gavage)	Y (Gavage)	Y (Gavage)
Vehicle	Y (Water)	Y (Water)	Y (Water)	Y (Water [j])	Y (Saline/Water [s])	Y (CMC-Na)	Y (CMC-Na)	Y (CMC-Na)
RCS-14 Total Score	10/14	9/14	10/14	11/14	9/14	12/14	12/14	11/14

Footnotes:

[h] Wang 2023 / Hu 2022 (Route): Administration was described as "oral/orally administered," without specifying a technical delivery term (e.g., gavage or intragastric).
[i] Yang 2025 (Route): Technical route not specified; animals were described as having "received" treatment

without a delivery term.
[j] Yang 2025 (Vehicle): Distilled water was explicitly reported as the control vehicle administered from days 8–21.
[k] Hu 2022 (Marker quantification): HPLC–UV chromatogram reports marker identification (5 markers), but numeric concentrations were not provided.
[l] Wang 2023 (Marker quantification): MS-based marker identification was reported (7 markers), but numeric quantification was not provided.
[m] Yang 2025 (Marker quantification): LC–MS identification was reported, but numeric concentrations were not explicitly listed.
[o] Wang 2025 (Fingerprint/markers): No batch-specific chemical fingerprinting or marker reporting was provided for the administered preparation.
[r] Zhao 2020 (Preparation): Preparation details (ratio/solvent/extraction) were referenced to Li et al. (2016).
[s] Wang 2025 (Vehicle): Sterile saline was reported for DSS/control groups; GQD was prepared in distilled water.
[t] Zhao 2020 (Marker quantification): Numeric marker values were explicitly printed in the chromatogram figure (e.g., baicalin 25.12%, puerarin 7.80%).
[u] Zhao 2021 (Marker quantification): Marker quantification was presented graphically without numeric tabulation.

Supplementary Table S3. Animal Source/supplier of included studies.

Study	Animal Source / Supplier	Location
Yang et al., 2025	Qizhen Experimental Animal Company	Hangzhou, China
Wang et al., 2023	Beijing Huafukang Biotechnology Co., Ltd.	Beijing, China
Xu et al., 2024	Cavens Experimental Animal Center	Changzhou, China
Wang et al., 2025	Guangdong Zhiyuan Biomedical Technology Co., Ltd	Guangdong, China
Hu et al., 2022	Vital River Laboratory Animal Technology	Hangzhou, China
Ruiyan Li et al., 2016	Model Animal Research Center of Nanjing University	Nanjing, China
Zhao et al., 2020	Changzhou Cavens Experimental Animal Limited Company	Changzhou, China
Zhao et al., 2021	Changzhou Cavens Experimental Animal Limited Company	Changzhou, China

Note: Supplier information was extracted as reported in each manuscript.

Supplementary Table S4. Detailed GRADE Evidence Profiles and Reasons for Downgrading

(A) Disease Activity Index (DAI)

Domain	Judgment	Rationale
Risk of bias (SYRCLE)	Downgrade 1	Nearly all studies showed unclear reporting in key methodological items (random sequence generation, allocation concealment, random housing, blinding of caregivers/assessors). While there was no systematic high risk, this limits confidence in internal validity.
Inconsistency (I^2)	No downgrade	Heterogeneity was moderate (I^2 = 43%), but the direction of effect was consistent across aqueous and ethanolic subgroups. Differences in magnitude appear attributable to dosing or experimental details rather than contradictory results.
Indirectness (preclinical)	No downgrade	Internal Comparability: All studies used comparable DSS-induced colitis models (2–3% DSS) with similar timing and DAI scoring criteria. No substantial differences were found that would compromise internal comparability.
Indirectness (To Humans)	Downgrade 1	Translatability: While the DSS model represents acute exacerbations, it is a chemically induced injury that does not fully reproduce the chronic, immune-mediated nature of human ulcerative colitis. Additionally, rodent doses/formulations are not directly translatable to practice.
Imprecision	No downgrade	The pooled effect for DAI was large with a relatively narrow 95% CI (–2.91 to –1.44) that did not cross the null. The total number of animals was adequate for preclinical evidence synthesis.
Publication Bias	Downgrade 1	Publication bias is considered likely in this field. The number of studies was insufficient for reliable funnel plot assessment. The preclinical field is characterized by a lack of protocol registration and limited publication of negative findings.
Upgrade Factors	No upgrade	Although the effect size was large (SMD –2.17), there was no clear or consistent dose–response pattern identified to justify upgrading.
FINAL CERTAINTY	LOW	The overall certainty is rated LOW due to methodological uncertainty (risk of bias), limited translatability to humans, and probable publication bias, despite a large and consistent effect size.

(B) Colon Length

Domain	Judgment	Rationale
Risk of bias (SYRCLE)	Downgrade 1	Most studies had unclear reporting in critical domains such as randomization, allocation concealment, and blinding. Although high risk was not consistently detected, the pervasive lack of methodological detail reduces confidence in internal validity.
Inconsistency (I^2)	Downgrade 1	Heterogeneity was very high ($I^2 = 88\%$), with considerable variability in the magnitude of the effect across studies despite the direction being consistently favorable. Differences in doses, extraction methods, disease severity, and study execution likely explain this heterogeneity.
Indirectness (preclinical)	No downgrade	Internal comparability: All included studies used DSS-induced colitis with comparable concentrations (2–3%) and measured colon length using standard procedures, supporting good internal comparability.
Indirectness (To Humans)	Downgrade 1	Translatability: Although DSS-induced colitis is widely accepted as a model of acute UC exacerbations, it does not reproduce the chronic, immune-mediated pathology of human UC. Moreover, the dosing and pharmacological preparation of GQD in rodents differ substantially from clinical formulations.
Imprecision	No downgrade	The pooled estimate for CL showed a relatively narrow confidence interval (0.91 to 1.45) and did not cross the null. The total number of animals was adequate for a preclinical synthesis, supporting precision.
Publication Bias	Downgrade 1	As with DAI, the limited number of studies, absence of preregistered protocols, and known tendency for positive results to be preferentially published in preclinical research suggest a high probability of publication bias.
Upgrade Factors	No upgrade	Although the effect size was consistently positive, no clear dose–response pattern was observed that would justify upgrading the certainty.
FINAL CERTAINTY	LOW	Final certainty was downgraded due to methodological limitations, very high heterogeneity, indirectness to humans, and likely publication bias, despite a precise and consistently favorable pooled effect.

(C) Histological Score

Domain	Judgment	Rationale
Risk of bias (SYRCLE)	Downgrade 1	Most studies showed unclear reporting for essential methodological items in the SYRCLE tool, including randomization, allocation concealment, random housing, and blinding. Although high risk was not dominant, the consistent lack of information limits confidence in study conduct.
Inconsistency (I^2)	No downgrade	Heterogeneity was moderate ($I^2 = 51\%$), but the direction of the effect was highly consistent across all studies, with variations mainly in magnitude. Differences likely reflect dosing and experimental conditions rather than conflicting effects.
Indirectness (preclinical)	No downgrade	Internal comparability: All included studies used chemically induced DSS colitis (2–3%) with comparable histological scoring methods, supporting good model uniformity and internal applicability.
Indirectness (To Humans)	Downgrade 1	Translatability: DSS colitis is widely used as a model of acute UC exacerbations, yet it does not fully capture the chronic, immune-driven nature of human ulcerative colitis. Additionally, differences in GQD preparation and dosing between rodent models and clinical use warrant downgrading.
Imprecision	No downgrade	The pooled effect for HS was large and the 95% CI did not cross the null (–4.12 to –1.93). The relatively narrow CI for an effect of this magnitude and the robust number of animals support high precision.
Publication Bias	Downgrade 1	The number of studies was insufficient to reliably assess funnel asymmetry, and the preclinical literature is known to preferentially publish positive findings, especially without preregistered protocols. Probable publication bias justifies downgrading.
Upgrade Factors	No upgrade	Although the effect size is very large (SMD –3.02), we did not observe a consistent or systematic dose–response pattern that would justify upgrading the certainty.
FINAL CERTAINTY	LOW	Final certainty was downgraded due to methodological limitations, partial translational applicability, and probable publication bias, despite a large and consistent therapeutic effect.

Supplementary Table S5. Full-text excluded studies and reasons for exclusion

#	Study (First author, Year)	Title	Journal	Reason for exclusion
1	Huang, 2024	Modified Gegen Qinlian Decoction Ameliorates DSS-Induced Ulcerative Colitis in Mice by Inhibiting Ferroptosis via Nrf2/GPX4 Pathway	Journal of Food Quality	Modified formula
2	Xu, 2025	A Comprehensive Review of Traditional Chinese Medicine in the Management of Ulcerative Colitis	American Journal of Chinese Medicine	Review
3	Xu, 2023	The mechanism of traditional medicine in alleviating ulcerative colitis: regulating intestinal barrier function	Frontiers in Pharmacology	Review
4	Li, 2025	Mechanism study on the treatment of ulcerative colitis by Gegen Qinlian nano-preparation through promoting M2 macrophage polarization	Frontiers in Molecular Biosciences	Different formula (nano-preparation)
5	Huang, 2025	Modified Gegen Qinlian Decoction Ameliorates DSS-Induced Colitis in Mice via the Modulation of NF-κB and Nrf2/HO-1 Pathways	Mediators of Inflammation	Modified formula
6	Lin, 2022	Gegen Qinliantang and Its Combinations Inhibit Oxidative Stress Injury in Ulcerative Colitis Rats by Regulating Nrf2/NQO1 Signaling Pathway	Chinese Journal of Experimental Traditional Medical Formulae	Non-mice
7	Huang, 2024	Exploring the immune landscape of disulfidptosis in ulcerative colitis and the role of modified gegen qinlian decoction in mediating disulfidptosis to alleviate colitis in mice	Journal of Ethnopharmacology	Modified formula
8	Wang, 2021	Modified Gegen Qinlian Decoction Regulates Treg/Th17 Balance to Ameliorate DSS-Induced Acute Experimental Colitis in Mice by Altering the Gut Microbiota	Frontiers in Pharmacology	Modified formula
9	Gao, 2016	Effect of administration temperature on efficacy of Gegen Qinlian Decoction on DSS-induced ulcerative colitis in rats	Chinese Traditional and Herbal Drugs	Non-mice

10	Wang, 2023	Modified Gegen Qinlian decoction ameliorated ulcerative colitis by attenuating inflammation and oxidative stress and enhancing intestinal barrier function in vivo and in vitro	Journal of Ethnopharmacology	Modified formula
11	Ma, 2023	Modified Gegen Qinlian decoction ameliorates DSS-induced chronic colitis in mice by restoring the intestinal mucus barrier and inhibiting the activation of γδT17 cells	Phytomedicine	Modified formula
12	Wang, 2023	Gegen Qinlian decoction (GQD) inhibits ulcerative colitis by modulating ferroptosis-dependent pathway in mice and organoids	Chinese Medicine	Incomplete information
13	Harwansh, 2024	Recent Insight into Herbal Bioactives-based Novel Approaches for Chronic Intestinal Inflammatory Disorders Therapy	Current Pharmaceutical Biotechnology	Review
14	Li, 2023	Natural products targeting Nrf2/ARE signaling pathway in the treatment of inflammatory bowel disease	Biomedicine & Pharmacotherapy	Review
15	Tang, 2025	Traditional Chinese Medicine formulas–based interventions on colorectal carcinoma prevention: The efficacies, mechanisms and advantages	Journal of Ethnopharmacology	Review
16	Li, 2022	Gegen Qinlian Decoction Alleviates Experimental Colitis and Concurrent Lung Inflammation by Inhibiting the Recruitment of Inflammatory Myeloid Cells and Restoring Microbial Balance	Journal of Inflammation Research	Different formula (dispensing granules)

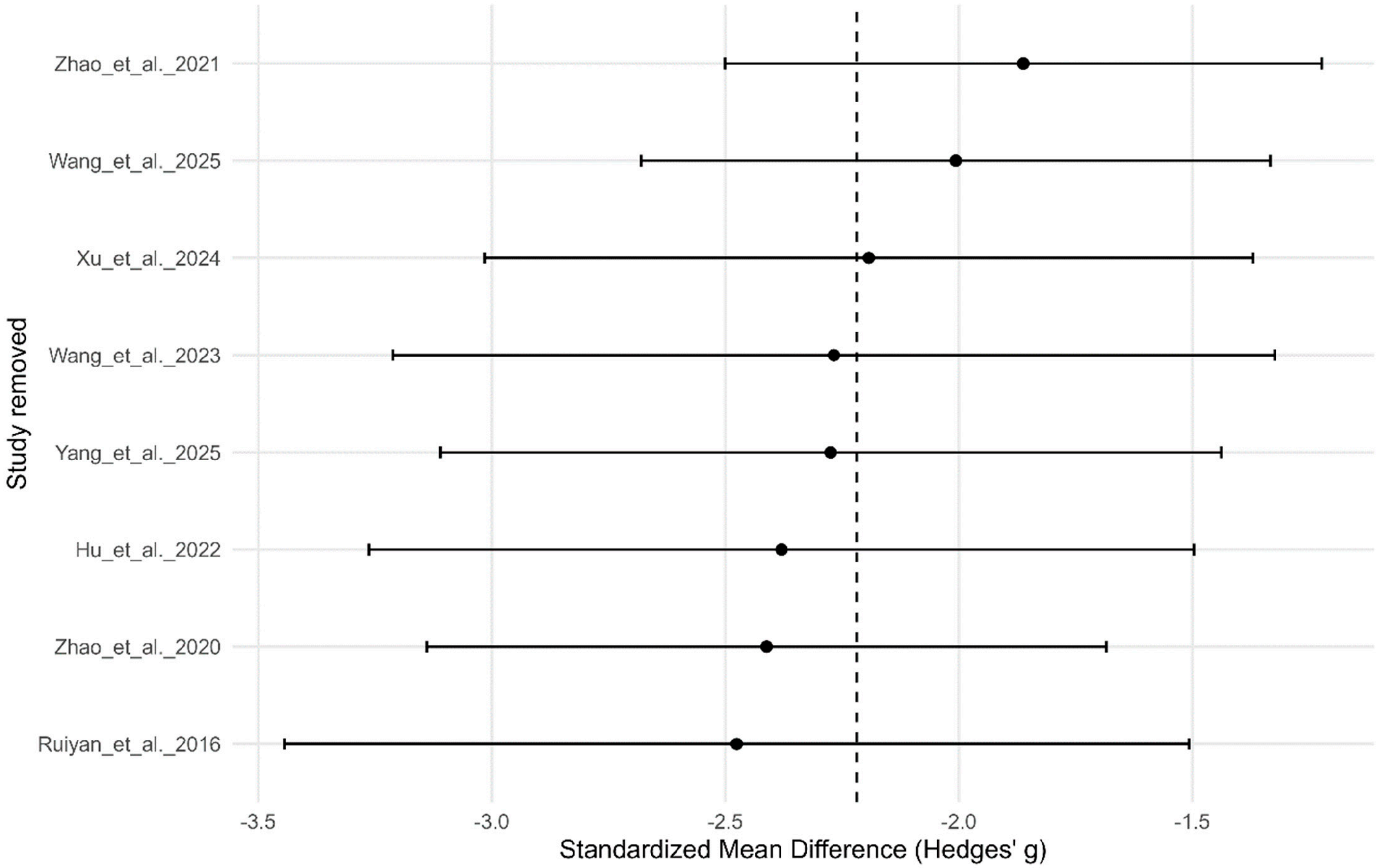

Figure S1. Leave-one-out sensitivity analysis (study level) for Disease Activity Index (DAI). Each point represents the recalculated pooled standardized mean difference (Hedges' g) after omitting one study, with horizontal bars indicating 95% confidence intervals. The vertical dashed line denotes the pooled estimate from the full model. The stability of the pooled effect across exclusions suggests that the overall DAI result was not driven by any single study.

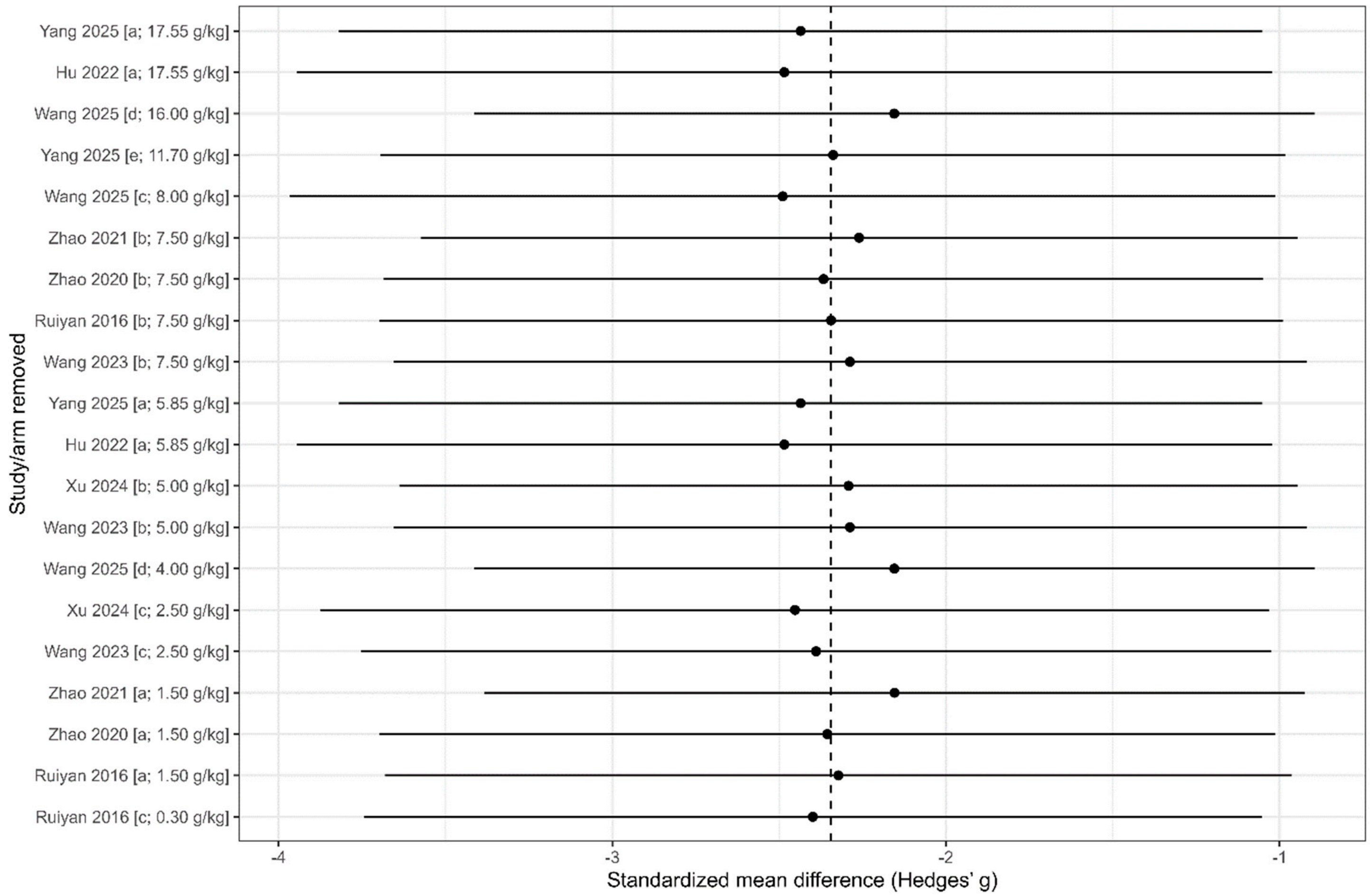

Figure S2. Leave-one-out sensitivity analysis (treatment-arm level) for Disease Activity Index (DAI) using a multilevel model. Each point represents the recalculated pooled standardized mean difference after omitting one extract–dose arm, with horizontal bars indicating 95% confidence intervals. The vertical dashed line denotes the overall multilevel estimate. The stability of the pooled effect across omitted arms supports robustness to multi-arm study structure and dose-arm variation.

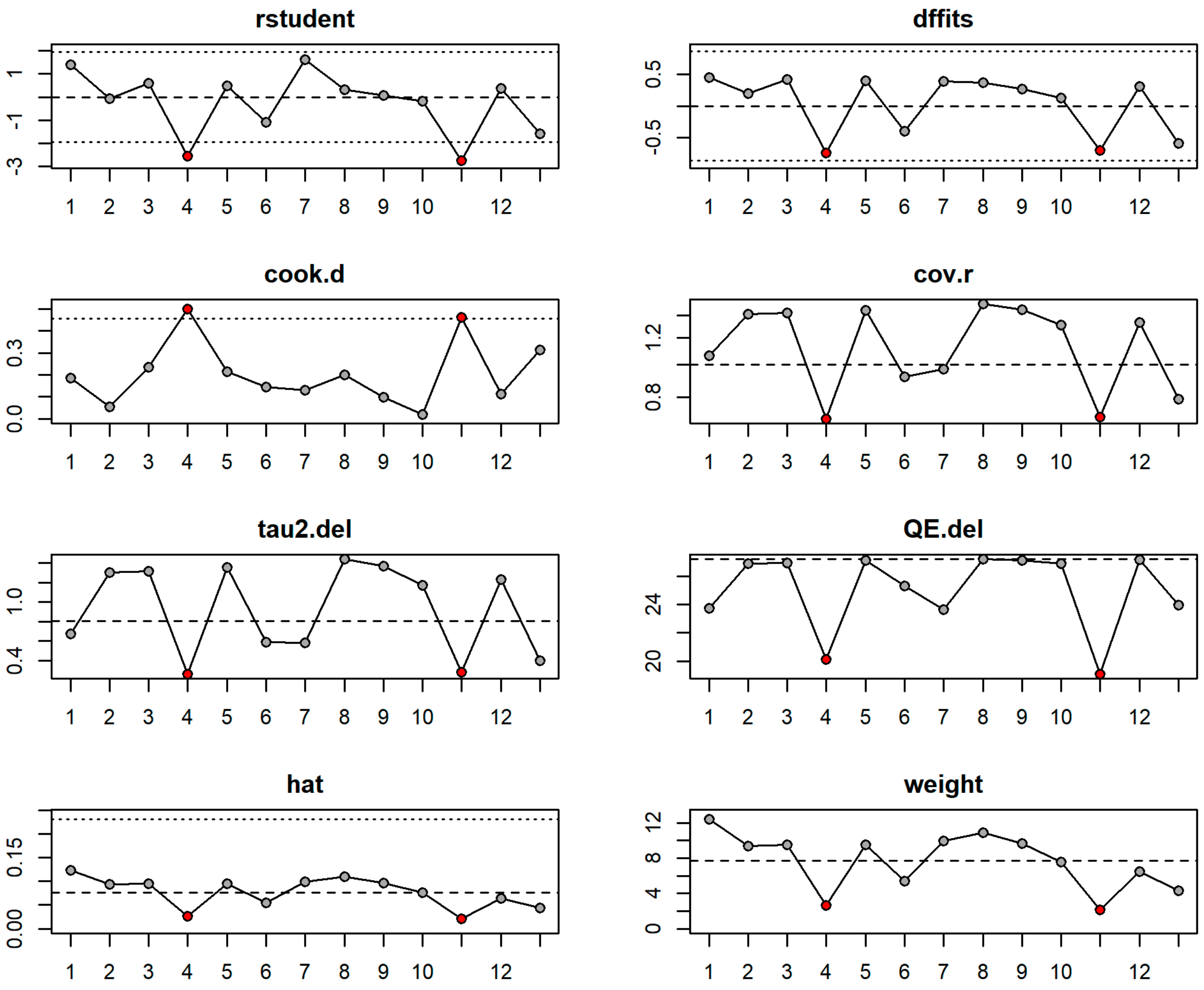

Figure S3. Influence diagnostics for the multilevel meta-analysis of Disease Activity Index (DAI). Panels display externally studentized residuals (*rstudent*), DFFITS, Cook's distance (*cook.d*), covariance ratio (*cov.r*), deletion diagnostics for between-study variance (τ^2.del) and heterogeneity (QE.del), leverage (*hat*), and model weights. Red points indicate comparisons flagged by the influence diagnostic routine. None of the flagged comparisons exceeded commonly used cutoffs across metrics, supporting the absence of undue influence from any single study or dose arm on the pooled estimate.

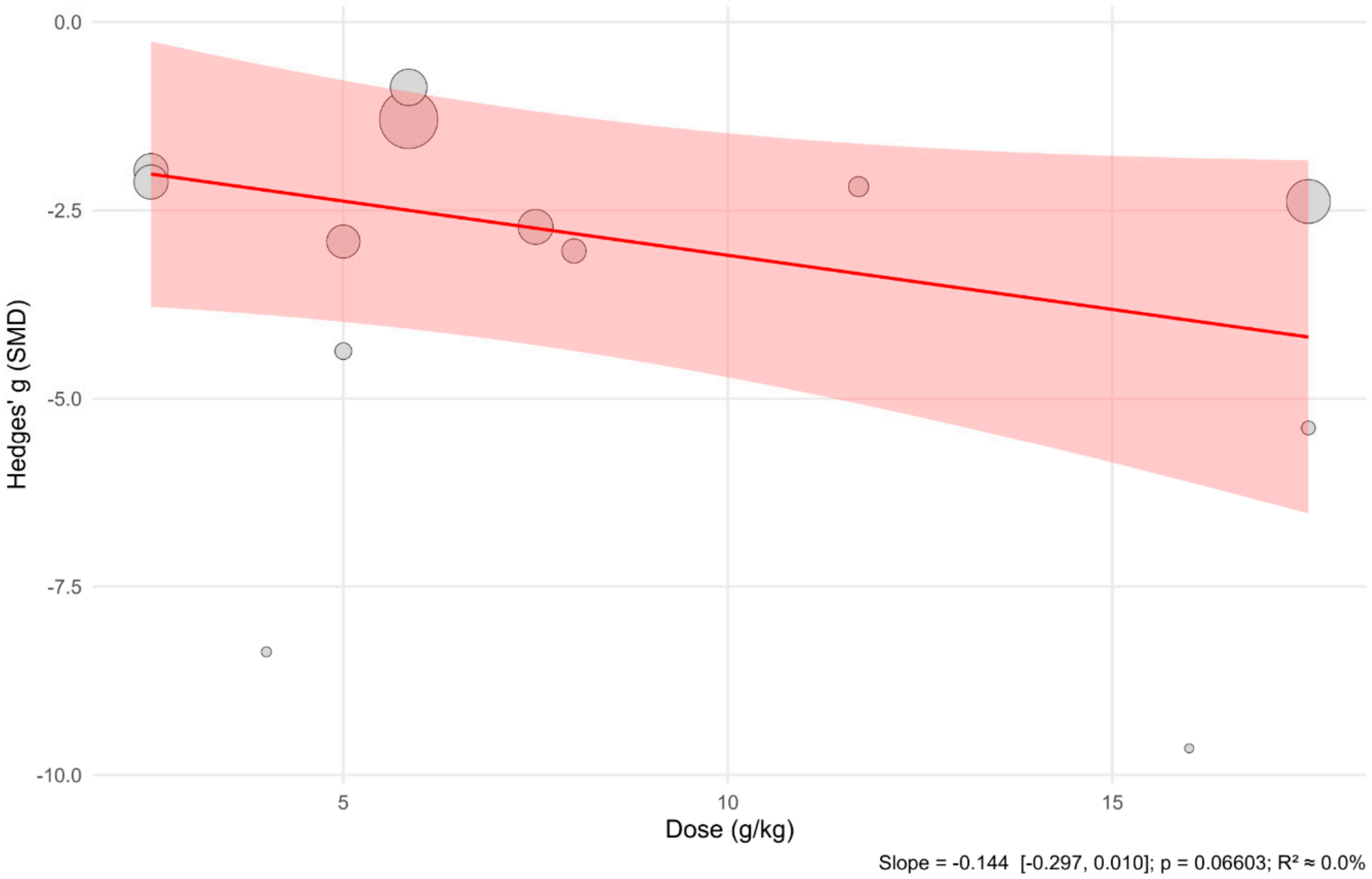

Figure S4. Dose meta-regression for aqueous Gegen Qinlian Decoction (GQD) on Disease Activity Index (DAI) effect sizes. A multilevel meta-regression was fitted using restricted maximum likelihood (REML) with Knapp–Hartung adjustment. Each point represents an individual treatment arm, with point size proportional to its inverse-variance weight. The solid line shows the estimated association between dose (g/kg) and standardized mean difference (Hedges' g), with the shaded band indicating the 95% confidence interval. The estimated slope suggested a non-significant trend toward larger effects at higher doses ($\beta = -0.144$; $p = 0.066$), and dose explained negligible heterogeneity ($R^2 \approx 0\%$). Dose was analyzed as reported (g/kg) within the aqueous subset; no conversion to crude-drug–equivalent units was performed.
